# Cs_4_Ca[Si_8_O_19_]: a new mixed tetrahedral–octahedral oxosilicate, its topological features and comparison with other interrupted framework silicates

**DOI:** 10.1107/S2052520625002537

**Published:** 2025-04-15

**Authors:** Volker Kahlenberg

**Affiliations:** a Institute of Mineralogy and Petrography University of Innsbruck Innrain 52 Innsbruck TyrolA-6020 Austria; Moscow State University, Russian Federation

**Keywords:** crystal structure, Cs_4_Ca[Si_8_O_19_], interrupted framework silicate, mixed tetrahedral-octahedral framework, topology, tilings

## Abstract

Single crystals of a previously unknown caesium calcium silicate with the composition Ca_4_Ca[Si_8_O_19_] have been obtained during a systematic study of the phase relations and compound formation in the system Cs_2_O–CaO–SiO_2_. Structure determination was based on a single-crystal diffraction data set recorded at 288 (2) K. The compound crystallizes in the monoclinic space group *P*2_1_/*n* and has the following basic crystallographic parameters: *a* = 7.1670 (6) Å, *b* = 12.0884 (10) Å, *c* = 12.4019 (10) Å, β = 90.044 (8)°, *V* = 1074.47 (15) Å^3^, *Z* = 2. The crystal structure was solved by direct methods. The sample showed twinning by pseudo-merohedry, which was accounted for in the subsequent least-squares refinements resulting in a residual of *R*1 = 0.036 for 1962 independent observed reflections and 149 parameters. The crystal structure of Cs_4_Ca[Si_8_O_19_] belongs to the group of interrupted framework silicates, in which the [SiO_4_] tetrahedra are linked in a three-dimensional network consisting of *Q*^4^ and *Q*^3^ groups in a 1:3 ratio. The linear backbones of the framework can be described as loop-branched *dreier* single chains. These ribbons are parallel to [100], and the translation period of about 7.2 Å along this axis reflects the periodicity of the chains. By sharing common corners, the condensation of these chains along the [001] direction leads to the formation of layers that are parallel to (010) and contain three- and nine-membered rings of tetrahedra. Alternatively, the crystal structure can be described as a mixed tetrahedral–octahedral framework between [SiO_4_] tetrahedra and [CaO_6_] octahedra containing cavities accommodating the caesium ions coordinated by seven and eight oxygen ligands, respectively. A detailed topological analysis of the mixed framework based on natural tiles is presented. Indeed, the net can be constructed from a total of only two different cages (tiles) having the following face symbols: [4^3^] and [3^4^.4^6^.6^2^.7^8^]. A comparison with related silicates containing [Si_8_O_19_] anions and already classified as well as hitherto unclassified interrupted frameworks is presented. Finally, the thermal expansion tensor has been determined in the temperature interval between 193 K and 288 (2) K.

## Abstract 2

The crystal structure of the mixed tetrahedral–octahedral framework silicate Cs_4_Ca[Si_8_O_19_] has been solved. A topological analysis of the present compound and other interrupted framework silicates is presented.

## 1. Introduction

Silicates based on [SiO_4_] tetrahedra (tetraoxosilicates) are of great significance within the Earth’s crust (Putnis, 1992 ▸) and simultaneously represent substantial constituents of numerous products related to applied mineralogy, including ceramics, cements, glasses, and refractories (Baumgart *et al.*, 1984 ▸; Taylor, 1997 ▸; Shelby, 2009 ▸). It is evident that they provide a compelling research opportunity for mineralogical crystallographers who are interested in structure–property relationships. Given this context, it is not surprising that the current version of the Inorganic Crystal Structure Database (ICSD) (Hellenbrandt, 2004 ▸) contains a plethora of natural and synthetic silicate structures.

A highly versatile and efficient classification system for the vast array of crystalline oxosilicates was developed by Friedrich Liebau and thoroughly outlined in his seminal textbook published forty years ago (Liebau, 1985 ▸). This classification emphasizes crystal chemistry aspects, particularly the manner in which the [SiO_4_] units are linked to each other. A subcategory of this system is framework silicates (tectosilicates), where the tetrahedra are connected into a three-dimensional network. Prominent natural representatives comprise the mineral groups of zeolites, feldspars, and feldspathoids. In the majority of the compounds belonging to this subgroup, including the aforementioned examples, the tetrahedra are quaternary (Q^4^), meaning that all four corners of a [SiO_4_] group are shared with adjacent tetrahedra. However, as Liebau previously noted, a small number of tectosilicates contain both quaternary *and* tertiary (Q^3^) units. Despite the increase in the number of these so-called interrupted framework silicates over the years, their total number remains limited when compared with the four-connected frameworks. Depending on the ratio of Q^4^ to Q^3^ units, different stochiometries of the silicate anions can be realized (see Table 1 ▸). The examples summarized in Table 1 ▸ refer to silicate frameworks without any structure-directing agents (template molecules) or organic cations in the cavities or tunnels of the nets. Moreover, no differentiation is made between the Si atoms and other tetrahedrally coordinated cations within the network, such as Be^2+^, B^3+^, Al^3+^, or Ge^4+^. Finally, frameworks containing secondary (Q^2^) units were also excluded.

It is noteworthy, that there are several representatives that possess a three-connected net based exclusively on tertiary tetrahedra. A comparison of the number of these particular entries with the group of phyllosilicates, which are also solely based on Q^3^ units and, therefore, exhibit the same Si:O ratio of 1:2.5, reveals once more that interrupted frameworks are the exception rather than the rule. On the other end of the scale of the Q^4^/Q^3^ ratios is the zeolite framework type *-iry*, which could be regarded as a highly diluted interrupted framework being close to four-connected. The chemical composition of the reference material, [Si_21.3_Ge_54.7_O_150_(OH)_4_], indicates that this phase is actually a silicogermanate rather than a germanosilicate.

Except for the Ge-containing nets with the highest Q^4^/Q^3^ ratios, all other phases summarized in Table 1 ▸ contain extra-framework cations consisting of large mono- or divalent cations, including Group 1 and Group 2 elements of the Periodic Table, Tl^+^, or Pb^2+^. The compound under investigation is a new member of the structurally interesting class of interrupted framework materials and simultaneously represents a new structure type.

In addition, structural information on caesium calcium silicates is generally limited or even non-existent. In contrast to the Na_2_O–CaO–SiO_2_ and K_2_O–CaO–SiO_2_ systems, whose phase and crystal chemistry have been extensively studied and for which numerous representatives are listed in the ICSD, the corresponding Cs_2_O–CaO–SiO_2_ system is largely unexplored. Until recently, the existence of such ternary silicates was not documented. The first caesium calcium silicate with a composition of Cs_2_Ca_4_Si_6_O_17_ was only discovered in 2025 (Kahlenberg, 2025 ▸). This imbalance in the number of studies is probably due to the significantly higher industrial significance of the first two oxide systems, which are of fundamental importance for the production of flat and hollow glass and for the formation of slag during biomass combustion and gasification (Shelby, 2009 ▸; Santoso *et al.*, 2020 ▸). The present investigation is part of an ongoing project to shed some light on the phase relationships and compound formation in alkali alkaline-earth silicates containing 1st main group elements of higher atomic number.

## 2. Experimental details

### 2.1. Single-crystal growth

Crystal growth experiments were based on mixtures of Cs_2_CO_3_ (Aldrich, 99.9%), CaCO_3_ (Merck, 99%), and SiO_2_ (Alfa Aesar, 99.995%) dried at 673 K in a box furnace to ensure that the reactants were free of physically adsorbed water. In addition, caesium carbonate is known to be hygroscopic. One gram of the starting reagents for a molar oxide ratio of Cs_2_O:CaO:SiO_2_ = 4:1:10 was weighed on an analytical balance and then homogenized in an agate mortar for 15 min. After mixing, the sample was transferred to a 50 ml platinum crucible which was covered with a lid. The container was heated from 294 K to 1373 K with a ramp of 2 K min^−1^. After annealing at the maximum temperature for 2 h, cooling was initiated at 0.1 K min^−1^ to 973 K where the crucible was removed after four days and quenched in air. The observed weight loss was approximately 1.0% greater than that expected from the disintegration of the carbonates, pointing to a small but measurable evaporation of the Cs_2_O component during the experiment. The melt cake was mechanically removed from the platinum container and further analyzed on a polarizing binocular indicating the presence of three distinct phases: (I) an isotropic glassy matrix with conchoidal fracture, (II) platy crystals of low optical quality showing undulous extinction between crossed polarizers and (III) smaller prismatic crystals with sharp extinction.

### 2.2. Single-crystal diffraction

Multiple samples of both crystalline phases were mounted on glass fiber tips using fingernail hardener and screened on an Oxford Diffraction Gemini R Ultra single-crystal diffractometer. The instrument is equipped with a four-circle kappa-goniometer and a Ruby CCD detector. To protect the crystals from potential hydration in air at 38% relative humidity (laboratory conditions), the diffraction experiments were performed in a dried air gas stream of 288 (2) K generated by an Oxford Cryosystems Desktop Cooler. The preliminary diffraction experiments confirmed the previous quality assessments based on optical microscopy. Phase II showed broad and partially smeared reflections, precluding the determination of even preliminary lattice parameters. Conversely, the diffraction spots of phase III were found to be sharp. Therefore, it was decided to focus the investigations on the latter compound. A full sphere of reciprocal space up to 25.35° θ was obtained with Mo *K*α radiation (see Table 2 ▸). The data were processed using the *CrysAlisPRO* software package (Rigaku Oxford Diffraction, 2020 ▸). After indexing, the diffraction pattern was integrated. Data reduction included Lorentz and polarization corrections. The sample was then cooled to 193 K to determine the lattice parameter at lower temperatures. Both data sets could be indexed with an orthorhombic unit cell with *a* ≃ 7.2 Å, *b* ≃ 12.1 Å and *c* ≃ 12.4 Å. After the correct chemical formula was established based on the structure determination (see Section 2.3), an analytical numeric absorption correction was applied to the data set using a multifaceted crystal model.

### 2.3. Structure solution, refinement and twinning

In the next step of data analysis, the reflections were merged in the orthorhombic Laue group *mmm*. The resulting internal residual *R*_int_ had a comparatively high value of 0.087. Furthermore, the observed systematic absences *h*00: *h* = 2*n* + 1, 0*k*0: *k* = 2*n* + 1, 00*l*: *l* = 2*n* + 1 and *h*0*l*: *h* + *l* = 2*n* + 1 were not compatible with an orthorhombic space group symmetry. Therefore, a possible symmetry reduction was considered. In particular, merging the data in the monoclinic Laue symmetry 12/*m*1 gave a much lower *R*_int_ value of 0.048. The combination of these findings pointed to the presence of a twinning by pseudo-merohedry (Parsons, 2003 ▸), wherein the reciprocal lattices of two monoclinic pseudo-orthorhombic cells are superimposed. Given the absence of any splitting of the reflections in precession-type reconstructions of reciprocal space, the corresponding monoclinic angle must be very close to 90°. Provided that the twinning hypothesis is true, the observed value for *R*_int_(*mmm*) indicates, that the volume fractions α and (1 − α) of the two twin domains in the crystal are significantly, but not extremely different from 0.5 each. Otherwise, the difference between *R*_int_(*mmm*) and *R*_int_(12/*m*1) would be much more pronounced.

**Figure 1:**
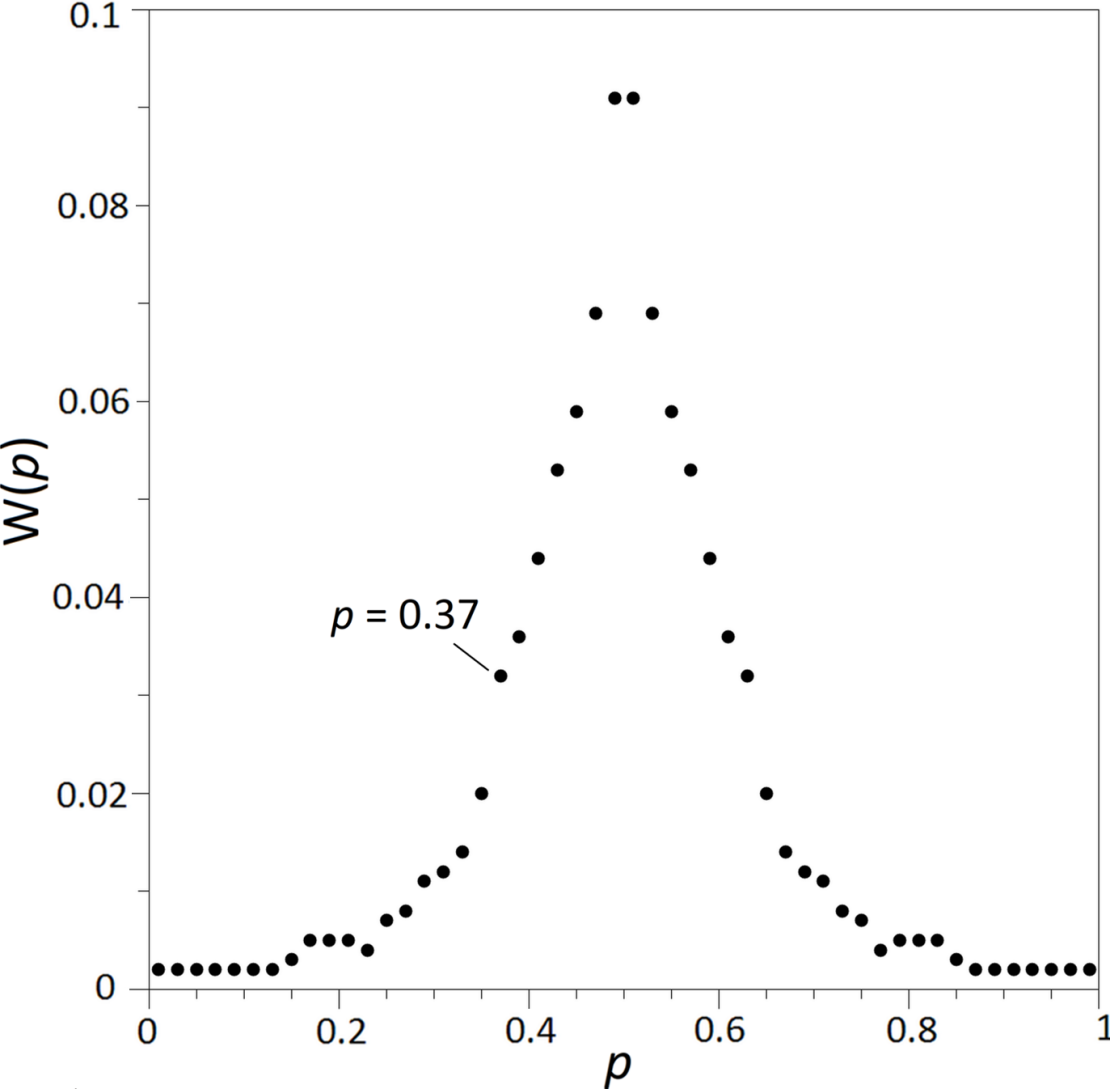
Relative frequency distribution W(*p*) for the twinned crystal of Cs_4_Ca[Si_8_O_19_]. The line indicates the volume fraction α of the smaller twin domain.

The existence of twinning by merohedry or pseudo-merohedry can be verified by statistical tests, under the assumption that α ≠ 0.5 (Kahlenberg, 1999 ▸). Due to the almost exact superposition of the reciprocal lattices of the different twin domains, the observed net intensity *I*_net_ of a reflection is the weighted sum of the intensities *I*_1_ and *I*_2_ of two reflections (*h*_1_*k*_1_*l*_1_) and (*h*_2_*k*_2_*l*_2_), respectively, superimposed by the twin law. The weighting factors are the volume fractions α and 1 − α of both twin domains, *i.e.I*_net_ = α*I*_1_ + (1 − α)*I*_2_. For each pair of twin-related reflections in the dataset, the ratio *p* = *I*_1_/(*I*_1_ + *I*_2_) can be calculated. According to Britton (1972 ▸), the relative frequency distribution of the ratio *p*, W(*p*), can be evaluated to detect the presence of twinning. In contrast with an untwinned crystal, where all possible values of *p* in the interval 0 ≤ *p* ≤ 1 can occur with a certain probability, the values of W(*p*) ≠ 0 for a twin are restricted to a region *p*_1_ ≤ *p* ≤ *p*_2_ symmetrical to *p* = 0.5. The values *p*_1_ and *p*_2_ of the discontinuities of W(*p*) correspond to the volume fractions α and 1 − α of the two twin individuals. To verify the twinning hypothesis, the program *TWIN3.0* (Kahlenberg & Messner, 2001 ▸) was employed, where the procedure after Britton is implemented as one test option. A twofold axis parallel [100] was assumed to be the twin element. The distribution W(*p*) derived for the actual data set is shown in Fig. 1 ▸. The form of the distribution confirms the hypothesis of the presence of twinning by pseudo-merohedry, and the volume fractions for the two twin-related orientations can be estimated to 0.37 and 0.63, respectively. Subsequently, data reduction was repeated to allow for a monoclinic distortion of the lattice. As expected, the deviations of β from 90° are very small (see Table 2 ▸).

The structure solution was successfully initiated using direct methods (*SIR2004*, Burla *et al.*, 2005 ▸) in space group *P*12_1_/*n*1. The resulting chemical formula of the compound derived after structure determination was Cs_4_Ca[Si_8_O_19_] with two formula units in the unit cell. A phase with this composition is not included in the currently available version of the ICSD. The initial model was deemed crystalchemically reasonable and was then optimized with full-matrix least-squares refinements using the *SHELXL-97* program (Sheldrick, 2008 ▸). The scattering curves and anomalous dispersion coefficients were obtained from the *International Tables for Crystallography*, Vol. C (Prince, 2004 ▸). The calculations with isotropic thermal displacement factors converged to *R*1 = 0.146. Extending the model to an anisotropic description of the thermal motion of the atoms increased the number of parameters from 63 to 148. However, the residual decreased only slightly (*R*1 = 0.121), and two silicon and two oxygen atoms in the asymmetric unit had non-positive definite thermal ellipsoids. When the above twin model was considered by introducing the twin law and volume fraction α of the smaller twin domain as an additional parameter, the calculations converged to *R*1 = 0.037. The largest shift/e.s.d. in the final cycles was < 0.001. Notably, α was refined to 0.376 (2), which is in excellent agreement with the value estimated from the a priori statistical test. Furthermore, the non-positive definite problems were resolved. Finally, an inspection of the fractional atomic coordinates using the ADDSYM algorithm implemented in the program *PLATON* (Spek, 2009 ▸) did not reveal any indication for unnecessarily low space-group symmetry. Table 3 ▸ lists the final coordinates and equivalent isotropic displacement parameters, while Table 4 ▸ provides selected interatomic distances and angles. Table 5 ▸ summarizes the anisotropic displacement parameters. Structural features were illustrated using the *VESTA3* program (Momma & Izumi, 2011 ▸). Bond valence sum (BVS) calculations have been performed with the program *ECoN21* (Ilinca, 2022 ▸) using the parameter sets of Brown & Altermatt (1985 ▸) for Ca–O and Leclaire (2008 ▸) for Cs–O interactions as well as Brese & O’Keeffe (1991 ▸) for the Si—O bonds. The corresponding results for all atoms are provided in the last column of Table 3 ▸. For the illustration of the three-dimensional representation surface of the thermal expansion tensor the program *WinTensor* (version 1.5) was employed (Kaminsky, 2014 ▸).

## 3. Results

### 3.1. Description of the structure

**Figure 2:**
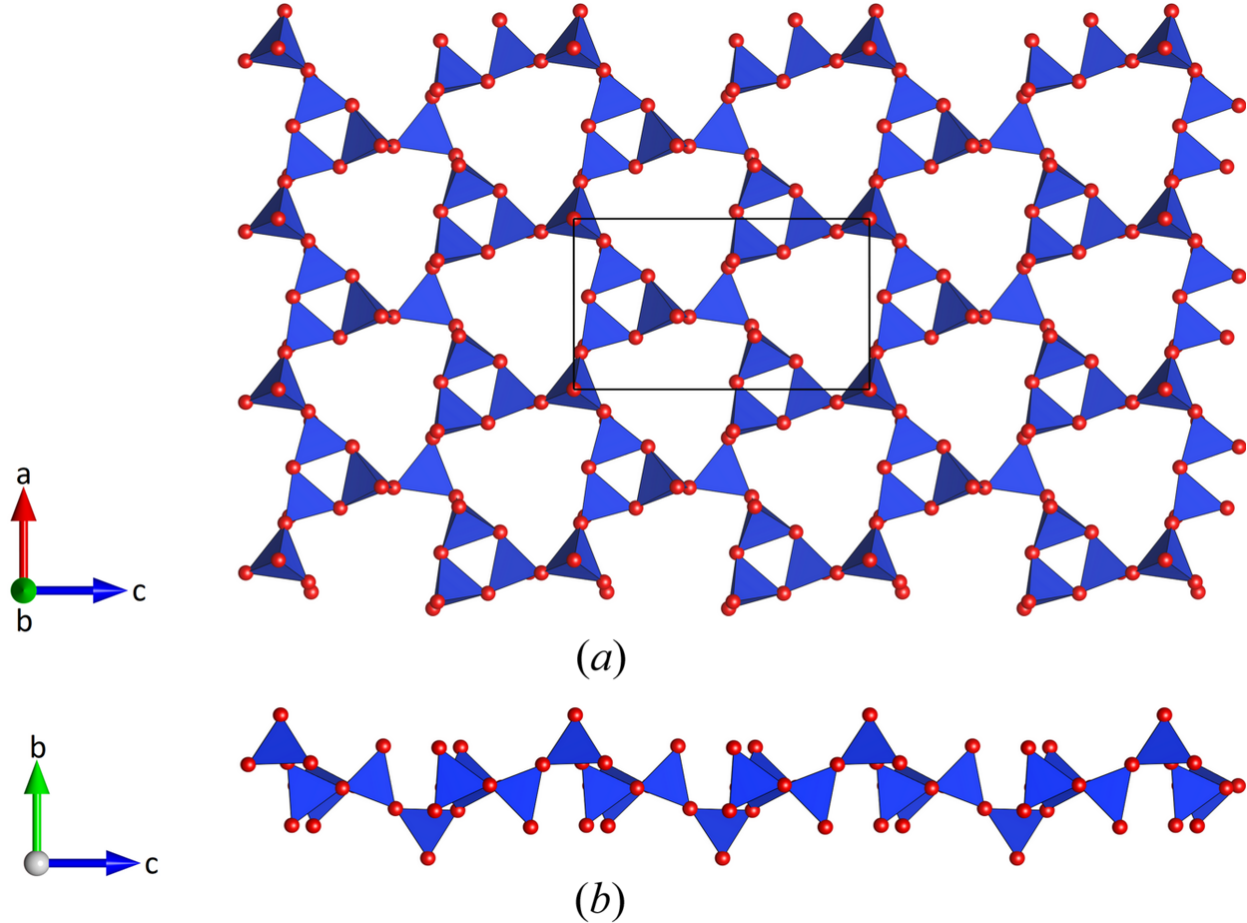
A single layer of [SiO_4_] tetrahedra in projections (*a*) perpendicular and (*b*) parallel to the sheet. Tetrahedra are shown in blue. Smaller red spheres represent the oxygen atoms.

The crystal structure of Cs_4_Ca[Si_8_O_19_] belongs to the group of interrupted framework silicates, in which the [SiO_4_] tetrahedra within the crystal are linked in a three-dimensional network consisting of Q^4^ and Q^3^ groups in a 1:3 ratio. This indicates, that the framework contains both bridging and non-bridging (nbr) oxygen atoms simultaneously. According to Liebau’s crystal chemical classification (Liebau, 1985 ▸), the linear backbones of the framework can be described as loop-branched *dreier* single chains and are slightly bended. These ribbons are parallel to [100], and the translation period of about 7.17 Å along this axis reflects the translation period of the chains [see Fig. 2 ▸(*a*)]. A common measure of the deviation of silicate chains from linearity is the stretching factor *f*_S_ (Liebau, 1985 ▸). It is defined as follows: *f*_S_ = *t*_c_ / (*l*_T_ × *P*), where *t*_c_ is the translation period along the chain, *l*_T_ is the length of the edge of a tetrahedron (both in Å), and *P* is the periodicity of the chain. A reference value of 2.7 Å has been proposed for *l*_T_, which is derived from the chains observed in the mineral shattuckite (Cu_5_[Si_2_O_6_(OH)]_2_), which have the most stretched chains observed (Liebau, 1985 ▸). For the individual single chains in Cs_4_Ca[Si_8_O_19_] (*P* = 3), a value of *f*_S_ = 0.885 is calculated.

By sharing common corners, the condensation of these chains along the [001] direction leads to the formation of layers that are parallel to (010). As illustrated in Fig. 2 ▸(*a*) and b, the corresponding sheets of silicate tetrahedra contain three- and nine-membered rings and do not exhibit a pronounced curvature. The sequence of directedness of up (**u**), down (**d**) and side (**s**) pointing tetrahedra in the rings is **sss** and **ussussdss** (or **dssdssuss**), respectively. The **sss** sequence of the tetrahedra residing on a local pseudo mirror plane is attributed to the pronounced repulsion of the tetravalent silicon cations in the sterically strained small three-membered rings.

**Figure 3:**
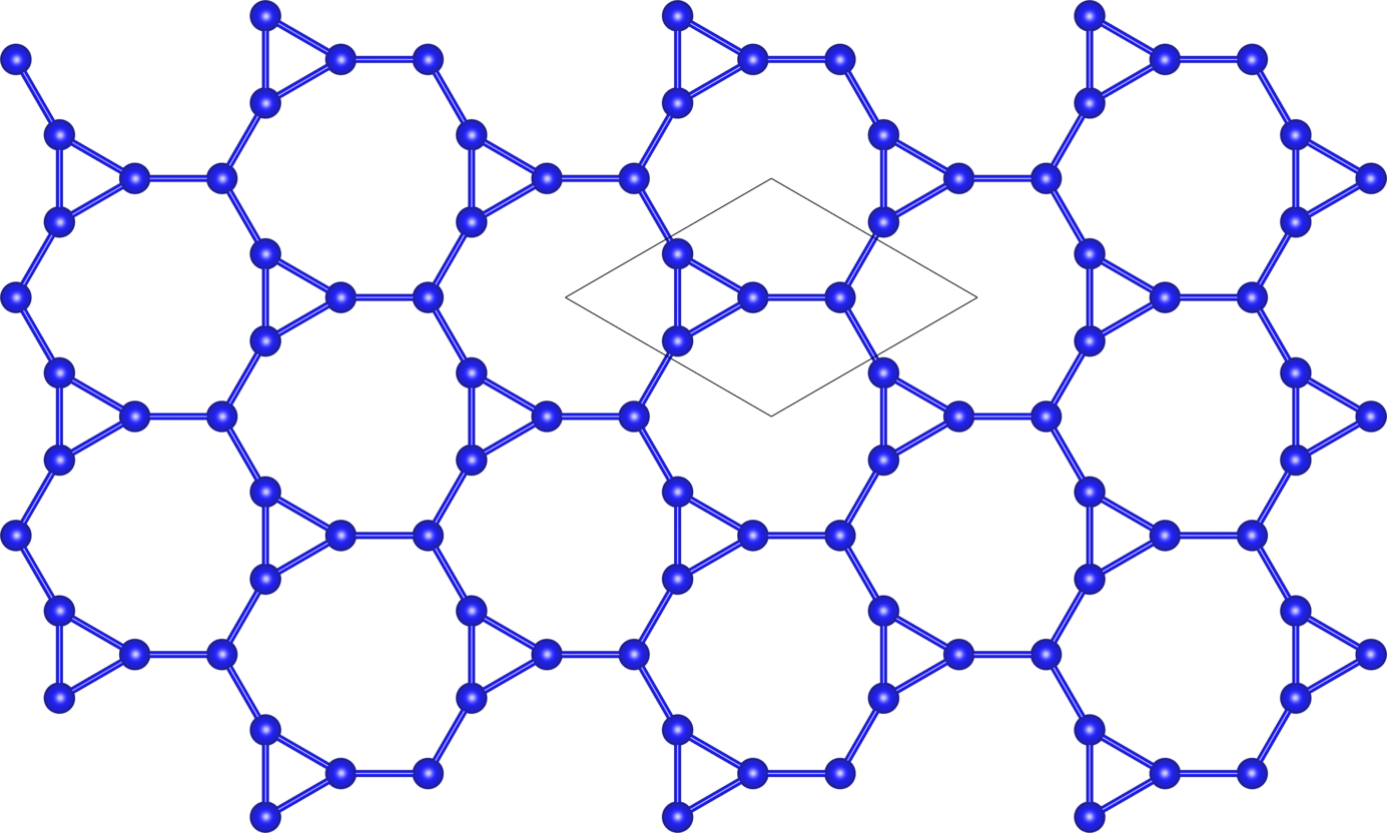
Idealized *hnb* net-type describing the connectivity of the Si-atoms within a single silicate layer of Cs_4_Ca[Si_8_O_19_]. The coordination sequences of the two different vertices (V1, V2) within this net-type are as follows: V1: 3-6-6-12-15-12-21-24-18-30 and V2: 3-4-8-12-11-18-19-18-28-26. The maximum topological symmetry of the *hnb* net is *p*3*m*1. The corresponding unit mesh is indicated.

Within a single sheet, a Si:O ratio of 1:2.5 is observed. The connectivity of the tetrahedra in the layer can be conveniently represented by a three-connected net, where the nodes denote the tetrahedra and the edges visualize the bonds between them. It is noteworthy, that two types of vertices can be distinguished, based on the number of the two different ring types that meet at a given node: (3.9^2^) and (9^3^), respectively. Using the nomenclature of Hawthorne (2015 ▸), where a subscript is introduced to account for the number of principally different nodes within a unit mesh, the net can be denoted as (3.9^2^)_6_(9^3^)_2_. A search in the Reticular Chemistry Structure Resource (RCSR) database (O’Keeffe *et al.*, 2008 ▸) revealed that the connectivity of the nodes within this net corresponds to the two-dimensional *hnb*-net type, which is shown in Fig. 3 ▸. The maximum symmetry of this planar net is described by the wallpaper group *p*3*m*1.

Adjacent sheets inside the unit cell are located at *y* = ¼ and *y* = ¾, respectively. They are linked by the oxygen atoms (O1) to form a three-dimensional silicate anion framework, the topological features of which will be described in more detail in the *Discussion*[Sec sec4].

The individual Si—O bond distances of the four symmetrically independent tetrahedra show a considerable scatter. Nevertheless, the observed values are in the normal range for silicate structures (Liebau, 1985 ▸). For the three Q^3^-type tetrahedra around Si2, Si3, and Si4, the Si—O bond distances to the non-bridging oxygen atoms are significantly shorter (1.549–1.558 Å) than the bridging Si—O bonds which range from 1.635 Å to 1.640 Å, respectively. The shortening of the terminal bond lengths compared with the bridging bond lengths results from the stronger attraction between the O and Si atoms than between the O atoms and the non-tetrahedral cations in the structure, and is a feature frequently observed for silicates. The values for the Si—O_nbr_ bond distances compare well with those observed in other interrupted frameworks such γ-Na_2_Si_2_O_5_ (Kahlenberg *et al.*, 2003 ▸) Tl_4_Si_5_O_12_ (Kahlenberg *et al.*, 2013 ▸) or K_4_CaSi_6_O_15_ (Karpov *et al.*, 1976 ▸). Conversely, the Q^4^-type tetrahedron around Si1 has fairly uniform Si—O bonds which average at about 1.597 Å. The O—Si—O bond angles range from 102.5° to 114.9°. These values are, again, rather typical of silicate structures. The distortion of the tetrahedra can be expressed numerically by means of the quadratic elongation (QE) and the angle variance (AV) (Robinson *et al.*, 1971 ▸). These parameters are summarized in Table 4 ▸. Not surprisingly, the Q^4^ tetrahedron around Si1 shows the least degree of distortion.

**Figure 4:**
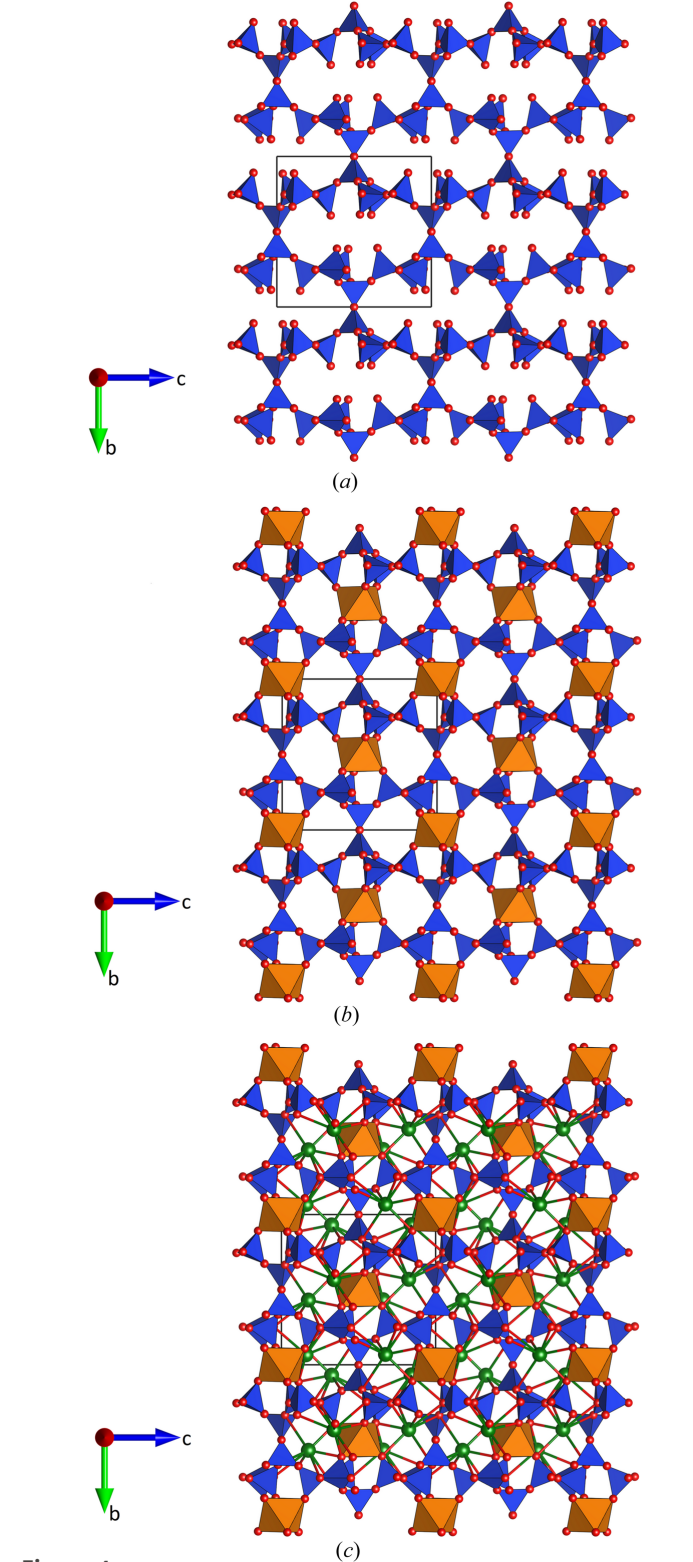
Projections parallel to [100] of (*a*) the interrupted tetrahedral framework, (*b*) the mixed tetrahedral–octahedral framework and (*c*) the whole crystal structure of Cs_4_Ca[Si_8_O_19_] are shown here. Octahedra around the Ca ions are presented in orange, while the Cs atoms are illustrated as larger green spheres. Cs—O bonds are indicated as well.

Most of the inter-tetrahedral bond angles are smaller than 140°, which is assumed to correspond to an unstrained Si–O–Si angle (Liebau, 1985 ▸). However, there is one exception. The Si1—O1—Si1 angle involving the oxygen atom that connects neighboring sheets has a value of 180 °. This straight angle is a direct consequence of the fact that O1 is located on a center of inversion, which also implies a staggered conformation of the two [Si1O_4_] tetrahedra which are linked into the [Si_2_O_7_] groups at the interface between the layers. For a long time, the existence of straight Si—O—Si angles has been controversially discussed in the literature. An excellent summary on this topic can be found in the paper of Baur & Fischer (2023 ▸). Notably, the thermal ellipsoid of O1 was not found to be disk-shaped and did not suggest a splitting of this oxygen position. Moreover, it can be excluded that the crystal structure of Cs_4_Ca[Si_8_O_19_] was refined in a space group with too high symmetry, thereby shifting certain oxygen atoms in positions of higher site-symmetry. It was therefore concluded that the value of 180° for the Si1—O1—Si1 angle is not an artifact. Linear Si–O–Si angles, for example, have also been reported in the interrupted framework of K_3_NdSi_7_O_17_ (Haile & Wuensch, 2000 ▸). A projection of the whole (3,4)-connected net of tetrahedra along [100] is shown in Fig. 4 ▸(*a*).

The single crystallographically independent calcium cation exhibits an octahedral coordination sphere with only minor deviations from regularity (see Table 4 ▸). The Ca ions in the barycenters are located at *y* = 0 and *y* = ½ and provide additional linkage between the two adjacent silicate sheets by forming bonds to three non-bridging oxygen atoms (O5, O8 and O9) from each layer. Consequently, a heteropolyhedral framework is formed [see Fig. 4 ▸(*b*)]. The remaining two caesium atoms are incorporated into the cavities of the framework, thereby balancing its negative charge and connecting the silicate layers. If the analysis is limited to Cs—O bonds with bond valences larger than 0.02 v.u., the caesium atoms are surrounded by eight (Cs1) and seven (Cs2) oxygen atoms, respectively. The corresponding average bond distances (see Table 4 ▸) are significantly larger than the mean values reported in LeClaire (2008 ▸) for Cs[7] (3.224 Å) and Cs[8] (3.245 Å) from a statistical analysis of literature data. Both CsO_*n*_ polyhedra are highly irregular, with the caesium atoms strongly shifted from the center to one side. A projection of the whole crystal structure of Cs_4_Ca[Si_8_O_19_] parallel to [100] is shown in Fig. 4 ▸(*c*).

### 3.2. Thermal expansion

The lattice parameters determined at 193 K had the following values: *a* = 7.1561 (6) Å, *b* = 12.0922 (10) Å, *c* = 12.3924 (9) Å, β = 90.036 (7) °, *V* = 1072.35 (15) Å^3^. In combination with the corresponding unit-cell metric at ambient conditions 288 K, see Table 2 ▸) the average thermal expansion tensor α_*ij*_ for the specific temperature interval was calculated from the thermal strain tensor ɛ_*ij*_ and the relationship α_*ij*_ = 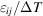 with the program *Win_Strain* (version 4.11; Angel, 2020 ▸). Using a finite Eulerian strain formalism referred to an orthonormal coordinate system {**x**, **y** and **z**} with **z** // **c**, **x** // a* and **y** = **z** × **x**, the following components of the 3 × 3 matrix for ɛ_*ij*_ were derived: ɛ_11_ = 0.0015 (1), ɛ_22_ = −0.0003 (1), ɛ_33_ = 0.0008 (1), ɛ_13_ = −0.00007 (9) = 0 within the accuracy of the calculations. With respect to the Cartesian coordinate system of the principal axes {**e**_1_, **e**_2_ and **e**_3_}, the following three principal strains are obtained: ɛ_1_ = −0.0003 (1), ɛ_2_ = 0.0008 (1) and ɛ_3_ = 0.0015 (1) indicating a pronounced anisotropy of the thermal strain. In fact, a small but measurable contraction upon heating is observed parallel to **e**_1_. Furthermore, the strain along **e**_3_ is five times larger than the magnitude of the strain parallel to **e**_1_. The resulting components of the average thermal expansion tensor α_*ij*_ in the temperature interval between 193 K and 288 K along the principal axes are as follows: −3 (1) × 10^−6^, 8 (1) × 10^−6^ and 16 (1) × 10^−6^ K^−1^.

## 4. Discussion

**Figure 5:**
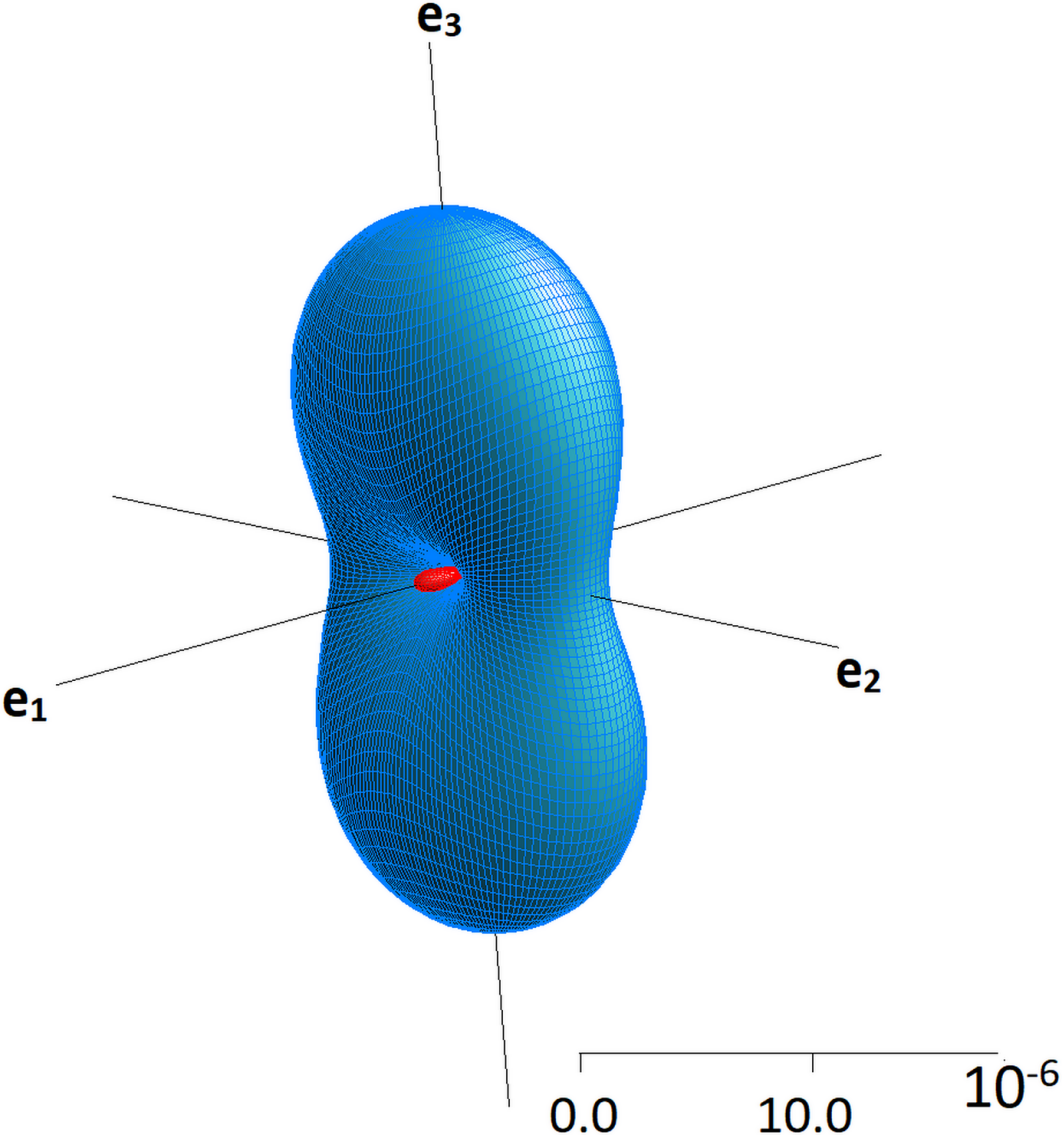
Side view of the representation surface of the thermal expansion tensor α_*ij*_ of Cs_4_Ca[Si_8_O_19_] for the temperature interval between 193 K and 288 K.The directions of the principal axes (eigenvectors) are shown. Blue and red colored parts of the surface represent regions of positive and negative values.

With the help of the symmetrical α_*ij*_ tensor, the relevant thermal expansion values can be calculated for any direction defined by a vector **q** whose three components are the direction cosines *q*_1_, *q*_2_ and *q*_3_, *i.e.* the cosines of the angles between **q** and the three axes of the Cartesian reference system. By plotting the individual values as a function of **q**, one obtains a geometric representation of the tensor in form of a surface in three-dimensional space. As illustrated in Fig. 5 ▸, the visualization of the corresponding surface provides concise information about the distribution of expanding and shrinking directions upon heating.

Using the aforementioned program *Win_Strain*, the following angles between the principal and the crystallographic axes {**a**, **b** and **c**} have been derived. The values given in parentheses refer to the corresponding angles with **a**, **b** and **c**, respectively: **e_1_**: (90°; 0°; 90°); **e**_2_: (95.2°; 90°; 174.8°); **e**_3_: (174.8°; 90.0°; 84.8°), that is, the contraction is along **b** while **e**_2_ and **e**_3_ are located within the **ac** plane. Finally, the components of the principal axes in the crystallographic coordinate system have been calculated and the corresponding vectors analyzed together with specific elements of the structure using the program *VESTA3* (Momma & Izumi, 2011 ▸). In particular, **e**_1_, which corresponds to the direction of the negative eigenvalue, is perpendicular to the tetrahedral layers. The direction of maximum thermal expansion (**e**_3_) during heating is almost parallel (deviation of 5°) with respect to the *dreier* single chains, while **e**_2_ is approximately perpendicular to the chains in the **ac** plane.

As shown in Table 3 ▸, the values for the bond valence sums of the crystallographically independent atoms indicate that the deviations between the calculated and the expected values, which correspond to the magnitudes of the formal charges of the cations and anions, are below 10%. However, larger positive or negative deviations are observed at the bridging oxygen atom O1 (BVS = 2.21 v.u.) and the Cs2 cation (BVS = 0.82). The latter phenomenon, also known as *underbonding*, suggests, that the framework cavity accommodating this particular Cs cation is slightly too large.

A computationally cost-effective method of assessing the relative stability of the structure is the so-called Global Instability Index (GII), as defined by Salinas-Sanchez *et al.* (1992 ▸). This index is calculated as the root mean square deviation of the bond valence sums from the oxidation states averaged over all cations and anions in the formula unit. According to Brown (2016 ▸), GII values exceeding 0.2 typically suggest the presence of an incorrect structure. For the phase under investigation, a GII index of 0.14 was determined, indicating a higher degree of steric strain. This finding may explain the observed sensitivity of the compound to hydration when exposed to a humid atmosphere of 38% RH for five days.

As mentioned above, the [SiO_4_] tetrahedra in Cs_4_Ca[Si_8_O_19_] are linked into a three-dimensional (3,4)-connected net. The framework density has a value of 14.9 *T* atoms/1000 Å^3^. In order to characterize this network in more detail, a topological analysis has been performed using the program *ToposPro* (version 5.4.3.0, Blatov *et al.*, 2014 ▸). For this purpose, the crystal structure has been described by a graph composed of the vertices (sites of the Si cations as well as O anions) and edges (bonds) between them. The nodes of the graph can be classified according to their *coordination sequences* {*N_k_*}. This number sequence represents a set of integers {*N_k_*} (*k* = 1,…,*n*), where *N_k_* is the number of sites in the *k*th coordination sphere of the respective atom that has been selected to be the central one (Blatov, 2012 ▸). The corresponding values for the four symmetrically independent Si sites up to *k* = 10 (without the oxygen nodes), as well as the cumulative numbers Cum_10_ including the central atoms, are listed in Table S1. Supplementary, the *extended point symbols* listing all shortest circuits for each angle for any non-equivalent Si- atom have been also determined. On the basis of the coordination sequences, three types of Si sites can be distinguished. The topological density, TD_10_, representing the rounded average of the Cum_10_ values for all central Si atoms in the asymmetric unit has a value of 546.

**Figure 6:**
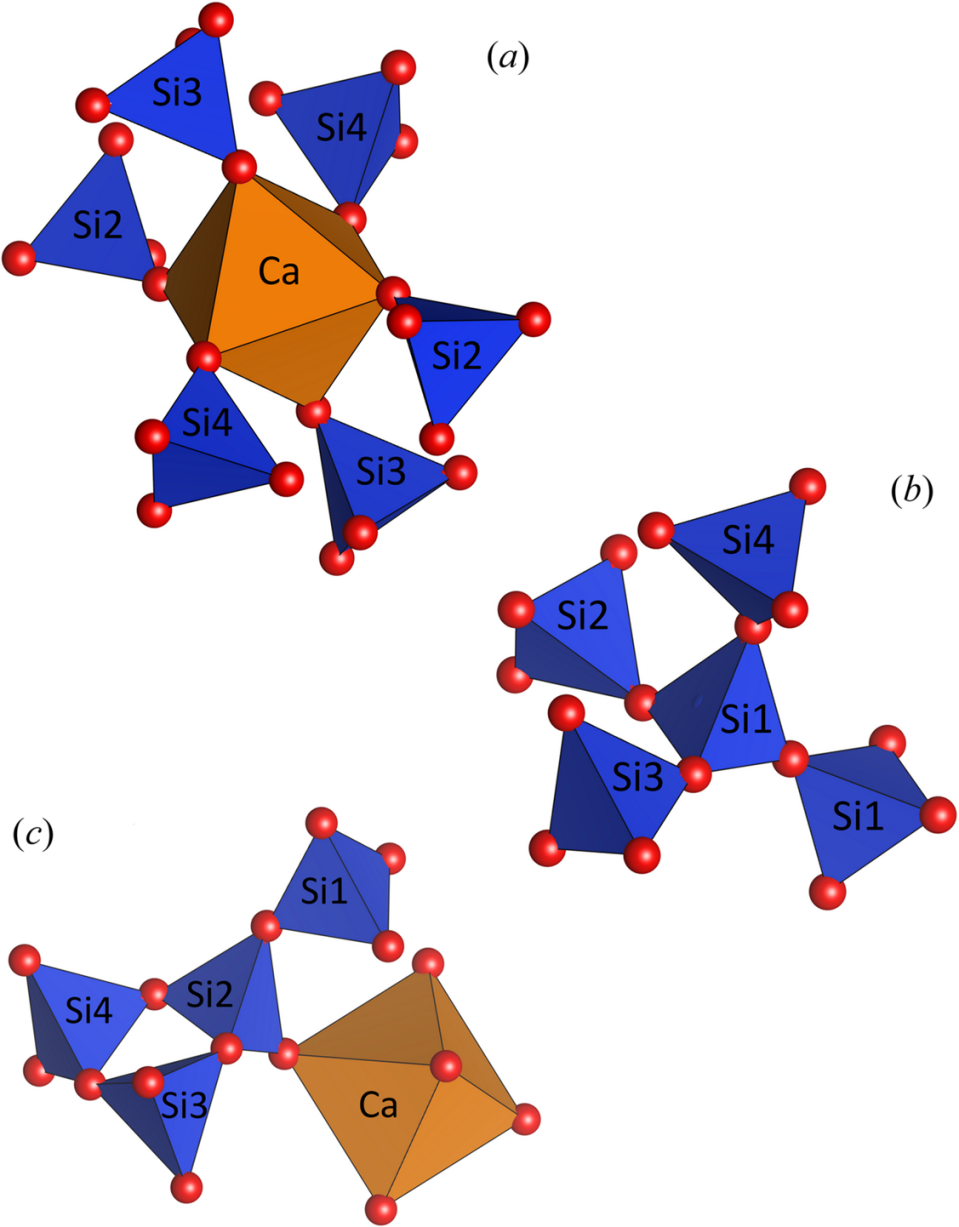
Polyhedral micro-ensembles (PMEs) in mixed tetrahedral octahedral framework of Cs_4_Ca[Si_8_O_19_]. (*a*) {6,6,18} (for Ca), (*b*) {4,4,12} (for Si1) and {4,4,13} (for Si2). The principal PMEs for Si3 and Si4 correspond to that of Si2. Octahedra and tetrahedra are shown in orange and blue, respectively.

An alternative understanding of the structure can be obtained by including the octahedra surrounding the calcium cations into the framework. In this case, the crystal structure of Cs_4_Ca[Si_8_O_19_] is regarded as a heteropolyhedral network. Using the aforementioned approach, the topological characteristics of this mixed tetrahedral–octahedral framework have been analyzed. In this model, the octahedral centers *M* (= Ca) of the octahedra now represent additional nodes of the net (see Table 6 ▸). The topological density TD_10_ was determined to be 1119. Furthermore, the polyhedral microensembles (PMEs) have been constructed. On the lowest sublevel they are formed for each octahedron and tetrahedron in the asymmetric unit by considering all directly bonded [CaO_6_] and [SiO_4_] groups. They represent a geometrical interpretation of the coordination sequences up to the index *k* = 3, when the oxygen atoms are included in the calculation. The PMEs of the first sublevel observed for the Ca nodes can be described as follows: each [CaO_6_] octahedron is immediately linked to six tetrahedra. Using the classification based on the calculation of the coordination sequences up to *k* = 3 (Ilyushin & Blatov, 2002 ▸) the PME of Ca can be denoted as {6,6,18}. The PMEs of the four crystallographically independent tetrahedral Si nodes conform to {4,4,12} (for Si1) and {4,4,13} (for Si2 to Si4), respectively [see Figs. 6 ▸(*a*) to 6 ▸(*c*)].

Another interesting aspect of the construction and classification of mixed tetrahedral–octahedral frameworks is to identify certain stable configurations of the *T* and *M* atoms that occur in different types of nets and, therefore, reflect transferable properties. These configurations are the so-called composite building units or CBUs (Liebau, 2003 ▸). In the literature, several types of CBUs have been proposed. One notable example is the natural building units (NBUs) (Blatov *et al.*, 2007 ▸), also known as natural tiles. A review of related terminology and definitions can be found in the paper by Anurova *et al.* (2010 ▸). A significant advantage of tilings of three-periodic nets based on natural tiles is the existence of a rigorous mathematical algorithm for their derivation. A natural tiling represents the minimum number of cages that cannot be split into smaller ones, forming a unique partition of space. Individual faces of the tiles (cages) are made from so-called essential rings (Blatov *et al.*, 2007 ▸). The concept of natural tilings has been applied to the current framework in Cs_4_Ca[Si_8_O_19_] using *ToposPro* and the results of the calculations are summarized in Table 7 ▸.

**Figure 7:**
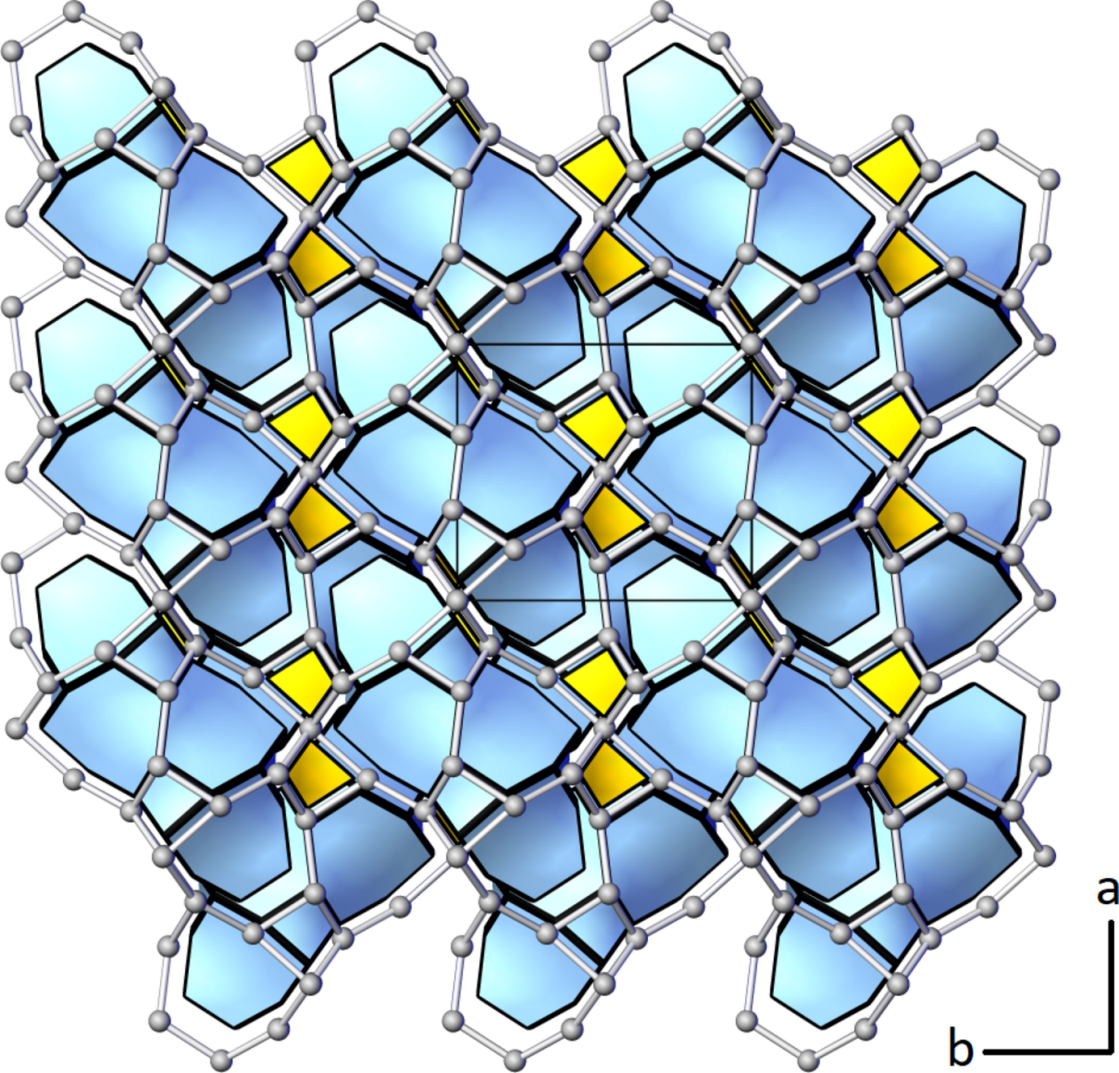
Arrangement of the two different natural tiles in the mixed tetrahedral–octahedral network of Cs_4_Ca[Si_8_O_19_] in a projection parallel to [001]. Gray spheres correspond to the nodes (*T* and *M* sites) of the net.

Two different natural tiles or cages can be distinguished by their face symbols (Blatov *et al.*, 2010 ▸) which encode the faces of which the tiles are made up. The general terminology is [*r^m^*.*s^n^*.*t^o^*…] indicating that a tile consists of *m* faces representing a polygon with *r* vertices, *n* faces forming a polygon with *s* corners, and so on. Notably, the present network involves one very simple tile [4^3^] with only five vertices in total as well as one more complex cage [3^4^.4^6^.6^2^.7^8^] with a total of 34 vertices. [4^3^] has been already observed as a NBU in zeolites such as natrolite or edingtonite (Baerlocher *et al.*, 2025 ▸). To the best of my knowledge the present [3^4^.4^6^.6^2^.7^8^] tile has not been described before. The arrangement of the natural tiles within the heteropolyhedral network is given in Fig. 7 ▸, which has been prepared using the program *3dt* (version 0.6.0, Delgado-Friedrichs, 2022 ▸).

The volumes of the two cages have values of 5.99 (for [4^3^]) and 525.25 Å^3^ (for [3^4^.4^6^.6^2^.7^8^]), respectively. The tiling signature and the transitivity of the tiling are also listed in the header of Table 7 ▸. The tiling signature enumerates all non-equivalent tiles written using their face symbols. The four integers defining the transitivity indicate that the present tiling has four types of vertices (first number), seven types of edges, five types of faces and two types of tiles (last number).

A number of the underlying nets associated with the interrupted silicate frameworks summarized in Table 1 ▸ have already been studied from a topological perspective and, as a result, have been included in one of the available online databases, for example, the Database of Zeolite Structures (Baerlocher *et al.*, 2025 ▸). For all other entries, a topological analysis was performed using the *TopCryst* (Shevchenko *et al.*, 2022 ▸) program, an internet accessible slimmed-down version of *ToposPro*. In many cases, the principal net types were observed for other crystalline materials such as metal organic frameworks (MOFs), but they were not yet linked with tetrahedral frameworks that are characteristic of oxosilicates. In these cases, the name or symbols of the corresponding nomenclature of the relevant database are listed in the last column of Table 1 ▸. The remaining interrupted frameworks, including Cs_4_Ca[Si_8_O_19_], represent previously unclassified network types, for which a full topological analysis was performed using the *ToposPro* program (see Table S1). The graphical representations of the corresponding natural tiles are given in Table S2. Obviously, these nets could be added as new entries to the respective internet resources. From the data presented in Tables S1 and S2, it is interesting to note, that the present compound is a rare example of an interrupted framework containing three-membered rings.

## 5. Conclusions

The majority of oxosilicate compounds based on [Si_8_O_19_]^6−^ anions belong to the group phyllosilicates (see Table 8 ▸). Notably, the mineral rhodesite listed in this table is also the namesake for a whole family of compounds, the so-called mero-plesiotype rhodesite series (Cadoni & Ferraris, 2010 ▸). A list of all 17 known natural and synthetic members of the series can be found in the corresponding table in Cadoni’s paper. The rhodesite group phases contain larger amounts of additional water molecules in the tunnel-like cavities enclosed by the silicate double sheets, which clearly distinguishes them from the anhydrous entries in Table 8 ▸.

Cs_4_Ca[Si_8_O_19_] represents the second example for an interrupted framework of silicate tetrahedra with a Si:O ratio of 1:2.375. The first occurrence of this anion type, with a stoichiometric formula of [Si_8_O_19_], has been reported in the crystal structure of the hydrous mineral thornasite (see Tables 1 ▸ and 8 ▸). However, the topologies (coordination sequences of the T-nodes, tilings, and tiling signatures) of the two frameworks are completely different (see Tables S1 and S2).

Regardless of the geometry of the anion type, a total of six charges are required to compensate for the negatively charged [Si_8_O_19_] units. When examining the *anhydrous* compounds listed in Table 8 ▸, it can be hypothesized that the number of *i* cations necessary for charge compensation, in combination with their specific coordination requirements / radii define the particular shape of the [Si_8_O_19_] anion that forms. For example, in Na_6_Si_8_O_19_ (*i* = 6), there is a large number of monovalent Na cations with comparatively low coordination numbers (5 and 6). Therefore, the slightly corrugated silicate layers are clearly separated by a dense layer of Na-centered octahedra and bipyramids, sharing four common edges each. In the anhydrous entries of Table 8 ▸ which form double layers, *i* = 4, there are always two larger alkali ions (K, Rb, Cs) and two smaller cations (Ca, Cu, V) with lower coordination numbers ([CaO_6_] octahedra, quadratic planar [CuO_4_] units, [VO_5_] pyramids, respectively) per silicate anion formula. The two single layers comprising a double layer show strong opposing curvatures enclosing tunnel-like cavities for the Group 1 elements with 10- to 12-fold coordination. Neighboring layers are connected by the polyhedral units of lower coordination number, which are linked into [Cu_2_O_6_] and [V_2_O_8_] dimers or [CaO_4_] chains. Linkage within these clusters/chains is due to edge-sharing. In the present compound, *i* has a value of 5 and there are four large Cs cations as well as only one smaller Ca ion located in the center of an octahedron. In the resulting framework, the [CaO_6_] octahedra are not condensed into larger polyhedral units, but rather share their six oxygen ligands only with the non-bridging atoms of the tetrahedral net. It should be noted, however, that the aforementioned hypothesis is based on a very limited data set and requires further validation through additional examples. Consequently, new synthesis experiments on alkali alkaline-earth silicates based on a [Si_8_O_19_] stoichiometry of the anion could prove a fruitful investigation.

Li *et al.* (2000 ▸) identified three main structural reasons that may cause disruptions of a tetrahedral silicate framework: (i) the presence of large cations (Ca^2+^, Na^+^) (ii) the presence of [BeO_4_]-tetrahedra in the framework, or (iii) the presence of larger (molecular) clusters in the channels. According to Table 1 ▸, larger substitutions of Si^4+^ by the considerably larger Ge^4+^ may also play a role. The present compound clearly belongs to the first group due to the large Cs^+^ ions.

Finally, what about the crystalline phase II mentioned earlier? Unfortunately, the quality of the crystals was insufficient for further crystallographic characterization. Moreover, the platy samples exhibited an even more pronounced hygroscopic behavior when compared to Cs_4_Ca[Si_8_O_19_], which precludes the preparation of the samples for EMP analysis using water as a polishing liquid. However, a reliable chemical composition is imperative for any tailored synthesis to yield better quality crystals or phase-pure polycrystalline material for an *abinitio* structure determination from powder diffraction data. In the laboratory in Innsbruck, a water-free polishing technique is currently established that addresses this issue. At any rate, this study unequivocally indicates, that there are additional phases of the system Cs_2_O–CaO–SiO_2_ awaiting their characterization.

## Supplementary Material

Crystal structure: contains datablock(s) global, I. DOI: 10.1107/S2052520625002537/yh5042sup1.cif

Structure factors: contains datablock(s) Cs4CaSi8O19. DOI: 10.1107/S2052520625002537/yh5042Isup2.hkl

Tables S1 and S2. DOI: 10.1107/S2052520625002537/yh5042sup3.pdf

CCDC reference: 2432464

## Figures and Tables

**Table 1:** Summary of several interrupted framework silicates containing only Q^3^ or Q^4^ and Q^3^ tetrahedra. In some of the examples, Si atoms in the tetrahedral centers have been partially replaced with Be, B, Al, or Ge. The last column gives the symbol of the underlying nets if it has been mentioned in one of the following available databases on three-dimensional periodic nets. EPINET: Euclidean Patterns In Non-Euclidean Tilings (Ramsden *et al.*, 2009 ▸); RCSR: Reticular Chemistry Structure Resource (O’Keeffe *et al.*, 2008 ▸); TTD: Topological Types Database (Alexandrov *et al.*, 2019 ▸); IZA-DSZ: Database of Zeolite Structures (Baerlocher *et al.*, 2025 ▸). For natural silicates, the mineral name is also given

Compound	*T*^4^:*T*^3^ ratio	*T*:O ratio	Reference	Net type
γ-Na_2_[Si_2_O_5_] (RT, HT)	0:1	1:2.5	Kahlenberg *et al.* (2003 ▸)	*lig* (RCSR)
Na[Si_2_O_4_(OH)]·H_2_O (grumantite)	0:1	1:2.5	Yamnova *et al.* (1989 ▸)	*lig* (RCSR)
K_2_[Si_2_O_5_]	0:1	1:2.5	DeJong *et al.* (1998 ▸)	*pcu-h* (RCSR)
K_8_Ca[Si_10_O_25_]	0:1	1:2.5	Kahlenberg *et al.* (2006 ▸)	*pcu-h* (RCSR)
K_2_Ce[Si_6_O_15_]	0:1	1:2.5	Karpov *et al.* (1976 ▸)	Bond sets: 2,3,4,5,6:*byl* (TTD)
K_4_Ca[Si_6_O_15_] (HT)	0:1	1:2.5	Liu *et al.* (2021 ▸)	*eth* (RCSR)
Na_2_[AlSi_3_O_8_(OH)] (ussingite)	1:1	1:2.25	Rossi *et al.* (1974 ▸)	3,4T1 (TTD)
Ca_6_[Al_5_Si_2_O_16_]Cl_3_ (wadalite)	1:1.333	1:2.286	Tsukimura *et al.* (1993 ▸)	*ctn* (RCSR)
Rb_6_[Si_10_O_23_]	1:1.5	1:2.3	Schichl *et al.* (1973 ▸)	*xci* (RCSR)
Cs_6_[Si_10_O_23_]	1:1.5	1:2.3	Lapshin *et al.* (2007 ▸)	*xci* (RCSR)
Ca_6_[Al_4_Si_6_O_23_](OH,H_2_O)_<2_[(Si,P)O_4_]_0.5_[(CO_3_,Cl)]_0.5_ (sarcolite)	1:1.5	1:2.3	Giuseppetti *et al.* (1977 ▸)	Unlisted
Na_2_[Si_3_O_7_]	1:2	1:2.333	Kahlenberg *et al.* (2002 ▸)	3,3,4T12 (TTD)
Na_2_[Si_3_O_7_]·H_2_O	1:2	1:2.333	Matijasic *et al.* (2000 ▸)	3,3,4T12 (TTD)
Cs_2_Er[Si_6_O_14_]F	1:2	1:2.333	Dabić *et al.* (2016 ▸)	Unlisted
Pb_4_Ca_2_[Si_9_B_3_O_28_] (khvorovite)	1:2	1:2.333	Pautov *et al.* (2015 ▸)	*sqc3881* (EPINET)
Na_12_Th_3_[Si_8_O_19_]_4_·18H_2_O (thornasite)	1:3	1:2.375	Li *et al.* (2000 ▸)	Unlisted
Cs_4_Ca[Si_8_O_19_]	1:3	1:2.375	This paper	Unlisted
Tl_4_[Si_5_O_12_]	1:4	1:2.4	Kahlenberg *et al.* (2013 ▸)	*pts-f* (RSCR)
K_3_Nd[Si_7_O_17_]	1:6	1:2.429	Haile & Wuensch (2000 ▸)	Unlisted
K_3_[Al_2_Si_4_O_12_(OH)] (lithosite)	2:1	1:2.167	Pudovkina *et al.* (1986 ▸)	*-lit* (IZA-DSZ)
Ca_4_[Be_3_AlSi_9_O_25_(OH)_3_] (bavenite)	2.25:1	1:2.154	Armstrong *et al.* (2010 ▸)	Unlisted
CaMn[Be_2_Si_5_O_13_(OH)_2_]·2H_2_O (chiavennite)	2.5:1	1:2.143	Tazzoli *et al.* (1995 ▸)	*-chi* (IZA-DSZ)
Ca_2_[Al_4_Si_4_O_15_(OH)_2_]·4H_2_O (parthéite)	3:1	1:2.125	Engel & Yvon (1984 ▸)	*-par* (IZA-DSZ)
Pb_7_Ca_2_[Al_12_Si_36_(O,OH)_100_·*n*(H_2_O,OH) (maricopaite)	5:1	1:2.083	Rouse & Peacor (1994 ▸)	Unlisted
Ba_4_Ca_6_[(Si,Al)_20_O_39_(OH)_2_](SO_4_)_3_·*n*H_2_O (wenkite)	9:1	1:2.05	Merlino (1974 ▸)	*-wen* (IZA-DSZ)
Na_6_[Si_16_Al_2_Be_2_O_39_(OH)_2_]·1.5H_2_O (leifite)	9:1	1:2.05	Coda *et al.* (1974 ▸)	Unlisted
[Si_115.2_Ge_44.8_O_312_(OH)_16_]	9:1	1:2.05	Jiang *et al.* (2011 ▸)	*-irt*
[Si_62.7_Ge_65.3_O_252_(OH)_8_]	15:1	1:2.031	Jiang *et al.* (2015 ▸)	*-ifu*
[Si_21.3_Ge_54.7_O_150_(OH)_4_]	20:1	1:2.026	Corma *et al.* (2010 ▸)	*-iry*

**Table 2:** Experimental details

Crystal data	
Chemical formula	Ca_4_Ca[Si_8_O_19_]
*M*_r_	1100.44
Crystal system, space group	Monoclinic, *P*2_1_/*n*
Temperature (K)	288
*a*, *b*, *c* (Å)	7.1670 (6), 12.0884 (10), 12.4019 (10)
β (°)	90.044 (8)
*V* (Å^3^)	1074.47 (15)
*Z*	2
Radiation type	Mo *K*α
μ (mm^−1^)	7.50
Crystal size (mm)	0.16 × 0.11 × 0.06

Data collection	
Diffractometer	Xcalibur, Ruby, Gemini ultra
Absorption correction	Analytical *CrysAlis PRO* 1.171.40.84a (Rigaku Oxford Diffraction, 2020). Analytical numeric absorption correction using a multifaceted crystal model (Clark & Reid, 1995 ▸)
*T*_min_, *T*_max_	0.45, 0.695
No. of measured, independent and observed [*I* > 2σ(*I*)] reflections	14449, 1962, 1775
*R*_int_	0.048
(sin θ/λ)_max_ (Å^−1^)	0.602

Refinement	
*R*[*F*^2^ > 2σ(*F*^2^)], *wR*(*F*^2^), *S*	0.036, 0.092, 1.06
No. of reflections	1962
No. of parameters	149
Δρ_max_, Δρ_min_ (e Å^−3^)	2.68, −0.90

Computer programs: *CrysAlis PRO* 1.171.40.84a (Rigaku OD, 2020 ▸), *SHELXL97* (Sheldrick, 2008 ▸).

**Table 3:** Atomic coordinates (×10^4^) and equivalent isotropic displacement parameters (Å^2^ × 10^3^) for Cs_4_Ca[Si_8_O_19_] *U*_eq_ is defined as one third of the trace of the orthogonalized *U*_*ij*_ tensor. Bond valence sum (BVS) values are given in valence units (v.u.).

Atom	Wyckoff-site	*x*	*y*	*z*	*U*_eq_	BVS
Cs1	4*e*	4962 (1)	669 (1)	8406 (1)	24 (1)	0.96
Cs2	4*e*	5109 (1)	4311 (1)	8253 (1)	30 (1)	0.82
Ca	2*a*	0	0	0	14 (1)	2.14
Si1	4*e*	37 (4)	3688 (2)	30 (2)	13 (1)	4.34
Si2	4*e*	2729 (3)	7699 (2)	8752 (2)	14 (1)	4.10
Si3	4*e*	3235 (4)	2290 (2)	784 (2)	14 (1)	4.15
Si4	4*e*	9566 (3)	2269 (2)	8027 (2)	14 (1)	4.15
O1	2*c*	5000	0	5000	10 (2)	2.21
O2	4*e*	4572 (8)	7467 (5)	9498 (5)	14 (2)	2.08
O3	4*e*	1637 (10)	2472 (5)	7498 (6)	16 (2)	2.08
O4	4*e*	2165 (9)	3345 (6)	228 (6)	28 (2)	2.13
O5	4*e*	9283 (11)	1084 (6)	8486 (6)	33 (2)	1.93
O6	4*e*	8050 (10)	2553 (5)	7088 (5)	17 (2)	2.13
O7	4*e*	8712 (10)	3330 (7)	1011 (5)	31 (2)	2.12
O8	4*e*	1869 (10)	8874 (6)	8891 (6)	29 (2)	1.92
O9	4*e*	2560 (10)	1145 (6)	379 (6)	30 (2)	1.94
O10	4*e*	9248 (10)	3268 (7)	8896 (6)	32 (2)	2.16

**Table 4:** Bond lengths (Å) and angles (°) for Cs_4_Ca[Si_8_O_19_] QE: quadratic elongation; AV: angle variance [see Robinson *et al.* (1971 ▸)].

Cs1—O9^i^	3.048 (7)	Cs1—O5	3.139 (8)
Cs1—O8^ii^	3.160 (8)	Cs1—O9^iii^	3.199 (8)
Cs1—O7^iv^	3.330 (7)	Cs1—O3	3.420 (7)
Cs1—O2^v^	3.456 (6)	Cs1—O6	3.572 (7)
〈Cs1—O〉	3.290		
Cs2—O8^vi^	3.059 (8)	Cs2—O5^vii^	3.072 (8)
Cs2—O10	3.319 (7)	Cs2—O6	3.324 (7)
Cs2—O4^i^	3.438 (7)	Cs2—O3	3.465 (7)
Cs2—O2^v^	3.528 (6)		
〈Cs2–O〉	3.315		
Ca—O9^ix^	2.346 (7)	Ca—O9	2.346 (7)
Ca—O5^iii^	2.347 (7)	Ca—O5^x^	2.347 (7)
Ca—O8^xi^	2.354 (6)	Ca—O8^xii^	2.354 (6)
〈Ca—O〉	2.359		
QE	1.000	AV	0.692
Si1—O1^viii^	1.5871 (19)	Si1—O10^x^	1.597 (7)
Si1—O4	1.599 (7)	Si1—O7^xiii^	1.603 (7)
〈Si1—O〉	1.597		
QE	1.003	AV	10.805
Si2—O8	1.558 (7)	Si2—O2	1.636 (6)
Si2—O3^xiv^	1.640 (8)	Si2—O7^xv^	1.643 (8)
〈Si2—O〉	1.619		
QE	1.006	AV	25.548
Si3—O9	1.549 (7)	Si3—O6^xvi^	1.635 (7)
Si3—O2^xv^	1.637 (6)	Si3—O4	1.640 (7)
〈Si3—O〉	1.615		
QE	1.006	AV	24.264
Si4—O5	1.555 (7)	Si4—O6	1.628 (7)
Si4—O10	1.635 (7)	Si4—O3^xvii^	1.641 (8)
〈Si4—O〉	1.615		
QE	1.005	AV	22.877
			
O9^ix^—Ca—O9	180.0	O9^ix^—Ca—O5^iii^	90.1 (3)
O9—Ca—O5^iii^	89.9 (3)	O9^ix^—Ca—O5^x^	89.9 (3)
O9—Ca—O5^x^	90.1 (3)	O5^iii^—Ca—O5^x^	180.0 (3)
O9^ix^—Ca—O8^xi^	90.7 (3)	O9—Ca—O8^xi^	89.3 (3)
O5^iii^—Ca—O8^xi^	88.8 (3)	O5^x^—Ca—O8^xi^	91.2 (3)
O9^ix^—Ca—O8^xii^	89.3 (3)	O9—Ca—O8^xii^	90.7 (3)
O5^iii^—Ca—O8^xii^	91.2 (3)	O5^x^—Ca—O8^xii^	88.8 (3)
O8^xi^—Ca—O8^xii^	180.0 (4)		
			
O1^viii^—Si1—O10^x^	106.9 (3)	O1^viii^—Si1—O4	106.2 (3)
O10^x^—Si1—O4	112.9 (4)	O1^viii^—Si1—O7^xiii^	106.1 (3)
O10^x^—Si1—O7^xiii^	111.9 (4)	O4—Si1—O7^xiii^	112.3 (4)
			
O8—Si2—O2	114.4 (4)	O8—Si2—O3^xiv^	111.6 (4)
O2—Si2—O3^xiv^	106.4 (3)	O8—Si2—O7^xv^	114.9 (4)
O2—Si2—O7^xv^	106.0 (4)	O3^xiv^—Si2—O7^xv^	102.5 (4)
			
O9—Si3—O6^xvi^	113.5 (4)	O9—Si3—O2^xv^	113.0 (4)
O6^xvi^—Si3—O2^xv^	105.6 (4)	O9—Si3—O4	114.4 (4)
O6^xvi^—Si3—O4	106.7 (4)	O2^xv^—Si3—O4	102.7 (3)
			
O5—Si4—O6	111.6 (4)	O5—Si4—O10	114.8 (4)
O6—Si4—O10	102.9 (4)	O5—Si4—O3^xvii^	113.7 (4)
O6—Si4—O3^xvii^	106.7 (3)	O10—Si4—O3^xvii^	106.3 (4)
			
Si1^xix^—O1—Si1^xviii^	180.0	Si2—O2—Si3^xv^	128.5 (4)
Si2^vi^—O3—Si4^xiii^	130.8 (4)	Si1—O4—Si3	135.4 (5)
Si4—O6—Si3^xix^	132.6 (5)	Si1^xvii^—O7—Si2^xv^	135.5 (5)
Si1^xx^—O10—Si4	139.9 (5)		

Symmetry codes: (i) *x*, *y*, *z* + 1; (ii) *x*, *y* − 1, *z*; (iii) −*x* + 1, −*y*, −*z* + 1; (iv) *x* − 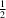, −*y* + 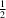, *z* + 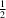; (v) −*x* + 1, −*y* + 1, −*z* + 2; (vi) −*x* + 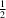, *y* − 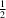, −*z* + 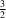; (vii) −*x* + 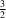, *y* + 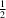, −*z* + 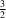; (viii) −*x* + 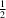, *y* + 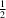, −*z* + 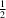; (ix) −*x*, −*y*,−*z*; (x) *x* − 1, *y*, *z* − 1; (xi) −*x*, −*y* + 1, −*z* + 1; (xii) *x*, *y* − 1, *z* − 1; (xiii) *x* − 1, *y*, *z*; (xiv) −*x* + 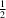, *y* + 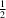, −*z* + 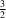; (xv) −*x* + 1, −*y* + 1, −*z* + 1; (xvi) *x* − 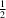, −*y* + 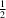, *z* − 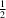; (xvii) *x* + 1, *y*, *z*; (xviii) −*x* + 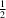, *y* − 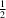, −*z* + 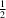; (xix) *x* + 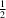, −*y* + 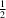, *z* + 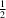; (xx) *x* + 1, *y*, *z* + 1.

**Table 5:** Anisotropic displacement parameters (Å^2^ × 10^3^) for Cs_4_Ca[Si_8_O_19_] The anisotropic displacement factor exponent takes the form: −2π^2^[*h*^2^a*^2^*U*_11_ + … + 2hka*b* *U*_12_].

Atom	*U*_11_	*U*_22_	*U*_33_	*U*_23_	*U*_13_	*U*_12_
Cs1	22 (1)	23 (1)	25 (1)	4 (1)	5 (1)	0 (1)
Cs2	34 (1)	30 (1)	27 (1)	3 (1)	−6 (1)	−1 (1)
Ca	16 (1)	11 (1)	16 (1)	0 (1)	6 (2)	1 (1)
Si1	14 (1)	10 (1)	16 (1)	1 (1)	0 (1)	1 (1)
Si2	7 (1)	25 (1)	11 (1)	−1 (1)	3 (1)	0 (1)
Si3	6 (1)	28 (2)	9 (1)	−1 (1)	−2 (1)	−1 (1)
Si4	11 (1)	26 (1)	5 (1)	1 (1)	0 (1)	−1 (1)
O1	11 (2)	5 (2)	14 (2)	0 (2)	−1 (2)	−1 (2)
O2	6 (4)	24 (3)	11 (3)	2 (2)	1 (2)	−3 (2)
O3	13 (4)	28 (4)	8 (3)	2 (2)	0 (3)	−2 (3)
O4	17 (4)	40 (4)	28 (4)	15 (3)	7 (3)	5 (3)
O5	37 (4)	31 (4)	30 (4)	16 (3)	−5 (3)	−6 (3)
O6	15 (4)	29 (4)	6 (3)	−2 (3)	−4 (3)	3 (3)
O7	28 (4)	52 (5)	14 (4)	1 (3)	2 (3)	−23 (4)
O8	24 (4)	34 (4)	30 (4)	−9 (3)	2 (4)	16 (3)
O9	22 (4)	35 (4)	33 (4)	−17 (3)	5 (3)	−13 (3)
O10	20 (4)	50 (5)	26 (4)	−20 (4)	−1 (3)	2 (3)

**Table 6:** Coordination sequences {*N_k_*} of the tetrahedrally (*T*: Si) and octahedrally (*M*: Ca) coordinated nodes (without the oxygen atoms), as well as the extended point symbols for Cs_4_Ca[Si_8_O_19_], when considered as a mixed tetrahedral–octahedral framework Cum_10_: cumulative numbers of the coordination sequence including the central node.

	Coordination sequences {*N_k_*} (*k* = 1–10)											
*T*/*M* atom	1	2	3	4	5	6	7	8	9	10	Cum_10_	Extended point symbols
Si1	4	10	22	46	66	101	148	175	229	311	1113	4.7.4.7.4.7_2_
Si2,Si3	4	10	25	43	71	107	133	195	241	286	1116	3.4_2_.6.7.7_2_.8_4_
Si4	4	10	26	41	69	111	128	193	238	282	1103	3.4_2_.7.7.7.7
Ca	6	14	24	58	74	98	166	186	232	322	1181	4.4.4.4.4.4.6.6.7.7.7.7.8_2_.8_2_.9_4_

**Table 7:** Summary of the tiling characteristics observed in the mixed tetrahedral–octahedral net of Cs_4_Ca[Si_8_O_19_] V: vertices; E: edges; F: faces. The color code refers to the color of the tiles in Fig. 7 ▸.

Tile 1	Tile 2
Tiling signature: 2 [4^3^]+[3^4^.4^6^.6^2^.7^8^]	Transitivity: [4.7.5.2]
Face symbol: [4^3^]	Face symbol: [3^4^.4^6^.6^2^.7^8^]
V, E, F: 5, 6, 3	V, E, F: 34, 52, 20
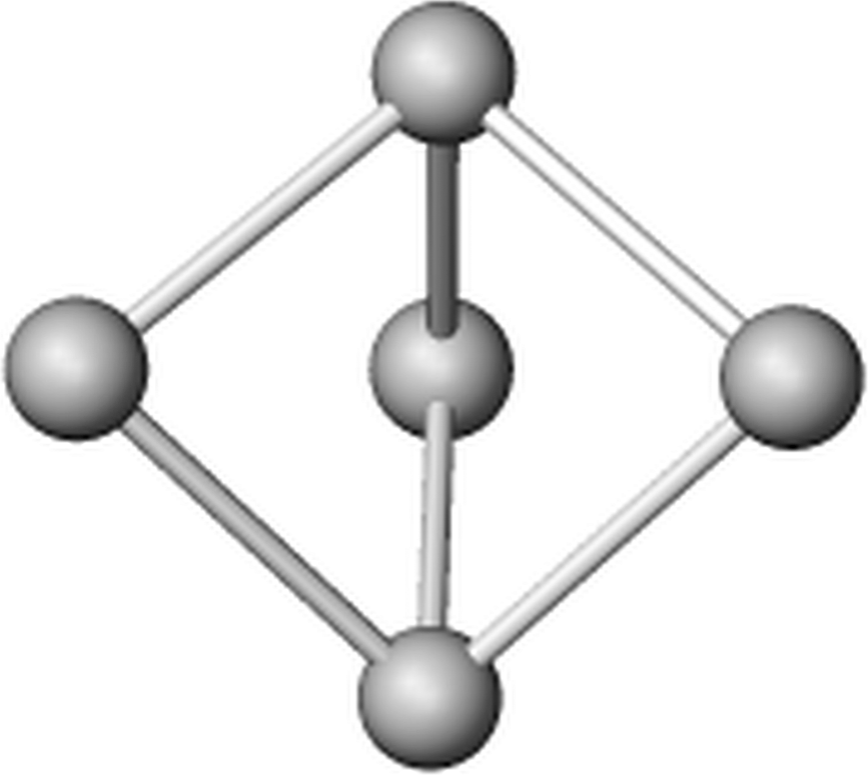	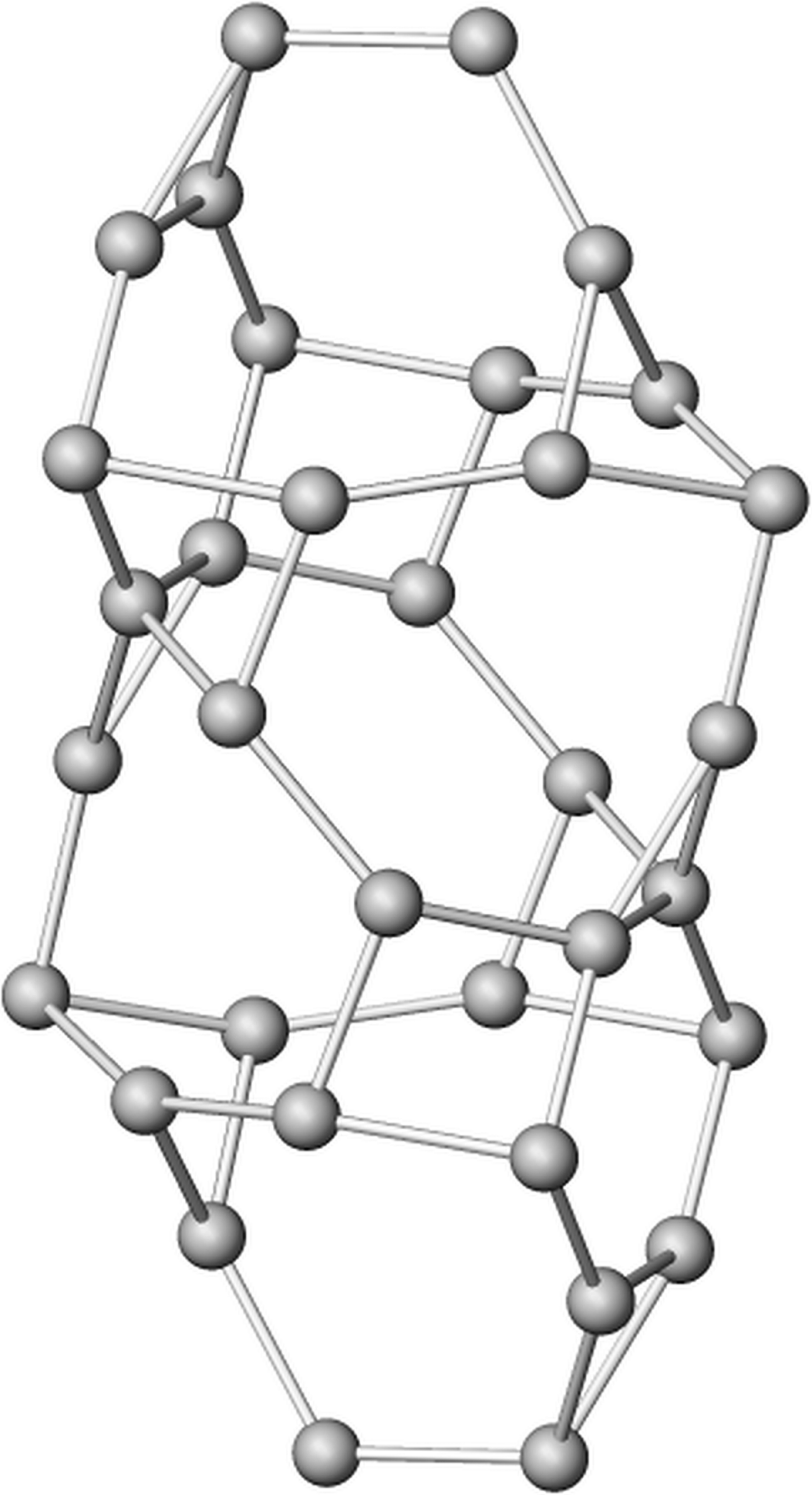
Color code: yellow	Color code: blue

**Table 8:** Summary of oxosilicates based on [Si_8_O_19_] anions

Compound	Anion type	Reference
Na_6_[Si_8_O_19_]	Single layer	Krüger *et al.* (2005 ▸)
Cs_4_Ca[Si_8_O_19_]	Interrupted framework	This paper
Na_12_Th_3_[Si_8_O_19_]_4_·­18H_2_O	Interrupted framework	Li *et al.* (2000 ▸)
K_2_Ca_2_[Si_8_O_19_]	Double layer	Schmidmair *et al.* (2017 ▸)
Cs_2_Cu_2_Si_8_O_19_	Double layer	Heinrich & Gramlich (1982 ▸)
Rb_2_(VO_2_)_2_[Si_8_O_19_]	Double layer	Prinz *et al.* (2008 ▸)
Rb_2_Cu_2_[Si_8_O_19_]	Double layer	Watanabe & Kawahara (1993 ▸)
KCa_2_[Si_8_O_18_(OH)]·­6H_2_O (rhodesite)	Double layer	Hesse *et al.* (1992 ▸)
