# Supplementary material for: Cs4Ca[Si8O19]: a new mixed tetrahedral–octahedral oxosilicate, its topological features and comparison with other interrupted framework silicates
Source: Acta Crystallogr B Struct Sci Cryst Eng Mater. 2025 Apr 15;81(Pt 3):325–36. doi: 10.1107/S2052520625002537 (PMC12147939; doi:10.1107/S2052520625002537)
Supplement: Supplementary file 3 [file b-81-00325-sup3.pdf]

STRUCTURAL SCIENCE
CRYSTAL ENGINEERING
MATERIALS

Volume 81 (2025)

Supporting information for article:

Cs4Ca[Si8O19]: A new mixed tetrahedral–octahedral oxosilicate, its topological features and comparison with other interrupted framework silicates

Volker Kahlenberg

Table S1 Topological characteristics of the tetrahedrally coordinated nodes (without the oxygen atoms) for the hitherto unclassified interrupted frameworks listed in Table 1. Cum_{10}: cumulative numbers of the coordination sequence including the central T-atom. TD_{10}: Topological density, rounded average of the Cum_{10} values. The T-node labels have been chosen in accordance with the relevant publications (see Table 1).

**$Cs_4CaSi_8O_{19}$**												
	Coordination sequences $\{N_k\}$ (k = 1–10)											
T-Atom	1	2	3	4	5	6	7	8	9	10	Cum_{10}	Extended point symbols
Si1	4	9	12	22	43	57	64	88	123	153	576	$8.9.8.9.9.11_2$
Si2, Si3	3	5	13	23	29	50	78	86	112	135	535	$3.8.9_2$
Si4	3	5	13	24	28	51	79	86	108	141	539	3.9.9
TD_{10}: 546												
Tiling signature: $[3^2.9^2.14^2]+[3^2.8^2.9^2.14^2]$						Transitivity: [4.7.4.2]						
Face symbol (Tile 1): $[3^2.9^2.14^2]$						Centroid (Tile 1): (0.50, 0.00, 0.00), Volume: 173.66 Å^3						
Face symbol (Tile 2): $[3^2.8^2.9^2.14^2]$						Centroid (Tile 2): (0.50, 0.50, 0.50), Volume: 363.57 Å^3						
**Sarcolite**												
	Coordination sequences $\{N_k\}$ (k = 1–10)											
T-atom	1	2	3	4	5	6	7	8	9	10	Cum_{10}	Extended point symbols
Al1	4	7	18	25	45	60	90	109	151	180	690	4.7.7.8.7.8
Si1	3	8	16	26	38	69	80	115	142	183	681	$7_2.7_2.8_2$
Si2	3	8	14	29	40	63	81	119	141	181	680	$4.7_2.8_2$
TD_{10}: 864												
Tiling signature: $2[7^4]+[8^6]+[4^8.7^8.8^6]$						Transitivity: [3.5.4.3]						
Face symbol (Tile 1): $[4^8.7^8.8^6]$						Centroid (Tile 1): (0.00, 0.00, 0.00), Volume: 835.27 Å^3						

Face symbol (Tile 2): $[7^4]$						Centroid (Tile 2): (0.50, 0.00, 0.25), Volume: 51.65 $Å^3$						
Face symbol (Tile 3): $[8^6]$						Centroid (Tile 3): (0.50, 0.50, 0.00), Volume: 245.87 $Å^3$						
**$Cs_2ErSi_6O_{14}F$**												
	Coordination sequences {N_k} (k = 1−10)											
T-Atom	1	2	3	4	5	6	7	8	9	10	Cum_{10}	Extended point symbols
Si1, Si3, Si4, Si5	3	6	13	24	35	48	69	94	118	145	556	$4.6_2.8_3$
Si2, Si6	4	10	16	22	36	56	77	96	119	150	587	$6.6_2.6.8.6.8$
TD_{10}: 566												
Tiling signature: $[6^3]+[4^2.6^3.8^2.14^2]$						Transitivity: [6.13.11.4]						
Face symbol (Tile 1): $[6^3]$ (t-kah)						Centroid (Tile 1): (0.25, 0.058, 0.25), Volume: 18.94 $Å^3$<br>Centroid (Tile 1): (0.00, 0.190, 0.249), Volume: 18.80 $Å^3$						
Face symbol (Tile 2): $[4^2.6^3.8^2.14^2]$						Centroid (Tile 2): (0.25, 0.875, 0.25), Volume: 367.95 $Å^3$<br>Centroid (Tile 2): (0.00, 0.125, 0.75), Volume: 364.06 $Å^3$						
**Thornasite**												
	Coordination sequences {N_k} (k = 1−10)											
T-Atom	*1*	2	3	4	5	6	7	8	9	10	Cum_{10}	Extended point symbols
Si1	3	6	11	19	32	51	72	95	121	150	561	$6.6.10_2$
Si2	3	6	11	19	32	51	72	93	116	150	554	$6.6.10_2$
Si3	3	7	12	19	33	53	70	89	123	154	564	$6.6.10_3$
Si4	3	6	11	20	33	51	73	95	118	149	560	6.6.10
Si5	4	8	13	22	36	52	72	99	129	158	594	$4.6.4.6.4.8_2$
Si6	4	9	16	23	34	54	76	101	130	159	607	$4.12_9.4.12_9.4.12_9$
TD_{10}: 569												
Tiling signature:						Transitivity: [6.10.8.6]						

$6[6.14^2]+6[10.14^2]+6[6.12.14^2]+[4^6]+3[4^2.6^2.12^2]$	
Face symbol (Tile 1): $[4^2.6^2.12^2]$	Centroid (Tile 1): (0.116, 0.00, 0.75), Volume: 130.92 Å^3
Face symbol (Tile 2): $[4^6]$ (t-cub)	Centroid (Tile 2): (0.00, 0.00, 0.00), Volume: 29.79 Å^3
Face symbol (Tile 3): $[6.14^2]$	Centroid (Tile 3): (0.149, 0.877, 0.085), Volume: 30.90 Å^3
Face symbol (Tile 4): $[6.12.14^2]$	Centroid (Tile 4): (0.092, 0.856, 0.698), Volume: 165.12 Å^3
Face symbol (Tile 5): $[10.14^2]$	Centroid (Tile 5): (0.333, 0.149, 0.917), Volume: 92.02 Å^3<br>Centroid (Tile 5): (0.333, 0.928, 0.917), Volume: 96.39 Å^3

$K_3NdSi_7O_{17}$

	Coordination sequences $\{N_k\}$ (k = 1−10)										
T-Atom	*1*	*2*	*3*	*4*	*5*	*6*	*7*	*8*	*9*	10	Cum_{10}	Extended point symbols
Si1, Si4	4	9	10	17	28	46	71	91	121	152	550	5.6.5.6.5.6
Si2	3	6	12	19	29	46	66	86	108	130	506	$5.12_2.12_2$
Si3, Si6	3	7	12	17	26	42	66	93	115	141	523	$5.6_2.9$
Si5	3	6	11	21	38	56	70	82	100	128	516	$6.12_2.12_2$

TD_{10}: 522	
Tiling signature: $2[6^3]+3[5^4.14^2]+3[6^2.12^2.14^2]+[6^2.12^6]$	Transitivity: [6.8.5.4]
Face symbol (Tile 1): $[5^4.14^2]$	Centroid (Tile 1): (0.00, 0.50, 0.50), Volume: 115.16 Å^3
Face symbol (Tile 2): $[6^3]$ (t-kah)	Centroid (Tile 2): (0.667, 0333, 0.151), Volume: 14.86 Å^3
Face symbol (Tile 3): $[6^2.12^2.14^2]$	Centroid (Tile 3): (0.00, 0.50, 0.00), Volume: 276.67 Å^3
Face symbol (Tile 4): $[6^2.12^6]$	Centroid (Tile 4): (0.00, 0.00, 0.00), Volume: 411.65 Å^3

Bavenite

	Coordination sequences $\{N_k\}$ (k = 1−10)											
T-Atom	*1*	*2*	*3*	*4*	*5*	*6*	*7*	8	9	10	Cum_{10}	Extended point symbols
T2(Be)	3	9	20	38	62	86	124	153	201	246	943	$6.6.6_2$
T1(Si)	4	8	18	38	66	94	114	160	200	252	955	$6.6.6.6.10_4.10_8$

T3(Be,Al,Si)	3	8	17	36	57	86	125	156	207	238	934	4.6.6
T4(Al,Si)	4	9	22	35	60	87	122	165	190	248	943	$4.6_2.6.6.8_4.8_4$
T5(Si)	4	12	20	38	60	89	120	152	204	247	947	$6.6.6_2.6_2.6_2.8_4$
T6(Si)	4	11	23	39	62	89	121	160	198	245	953	$6.6.6.6_2.6.6_2$
TD_{10}: 946												
Tiling signature: $[4^2.8^2]+2[6^4]+[6^{16}.8^2]$							Transitivity: [6.7.6.3]					
Face symbol (Tile 1): $[4^2.8^2]$ (t-kds)							Centroid (Tile 1): (0.00, 0.00, 0.00), Volume: 24.87 Å^3					
Face symbol (Tile 2): $[6^{16}.8^2]$							Centroid (Tile 2): (0.00, 0293, 0.25), Volume: 457.71 Å^3					
Face symbol (Tile 3): $[6^4]$ (t-hes)							Centroid (Tile 3): (0219, 0.50, 0.00), Volume: 37.55 Å^3					
**Maricopaite**												
	Coordination sequences $\{N_k\}$ (k = 1−10)											
T-Atom	*1*	*2*	*3*	*4*	5	6	7	8	9	10	Cum_{10}	Extended point symbols
Si1, Si2	4	12	19	34	58	85	108	144	187	231	883	$5.5.5.5_2.5.7$
Si3,Si6	4	11	18	30	53	88	110	135	182	237	869	$5.5.5.5_2.6.7$
Si4, Si5	3	9	19	31	49	79	107	138	176	221	833	$5.5.5_2$
Si7, Si8	4	11	24	37	55	83	117	146	183	235	896	$5.8.5.8.5_2.10_2$
TD_{10}: 872												
Tiling signature: $2[5^4]+[5^6.8.12^2]+[5^{10}.8.12^2]$							Transitivity: [8.16.14.6]					
Face symbol (Tile 1): $[5^4]$ (t-tes)							Centroid (Tile 1): (0.284, 0.321, 0.50), Volume: 24.07 Å^3<br>Centroid (Tile 1): (0.216, 0.186, 0.00), Volume: 24.39 Å^3					
Face symbol (Tile 2): $[5^6.8.12^2]$ (t-mor)							Centroid (Tile 2): (0.00, 0.474, 0.50), Volume: 314.88 Å^3<br>Centroid (Tile 2): (0.00, 0.533, 0.00), Volume: 314.48 Å^3					
Face symbol (Tile 3): $[5^{10}.8.12^2]$							Centroid (Tile 3): (0.50, 0.398, 0.00), Volume: 357.36 Å^3<br>Centroid (Tile 3): (0.00, 0.109, 0.50), Volume: 359.47 Å^3					
**Leifite**												

	Coordination sequences $\{N_k\}$ (k = 1–10)										
T-Atom	1	2	3	4	5	6	7	8	9	10	Cum_{10}	Extended point symbols
Be1	3	9	24	39	74	102	146	195	224	312	1129	$7_2.7_2.7_2$
Si1	4	12	27	45	74	104	142	186	238	291	1124	5.6.6.6.6.6
Si2	4	11	24	44	75	100	142	192	227	298	1118	$4.6_2.5.5.7_2.7_2$
Si3	4	10	23	46	70	108	148	184	251	287	1132	$4.8_2.5.7_2.5.7_2$

TD_{10}: 1125	
Tiling signature: $6[6.7^2]+[6^8]+2[4^3.5^6.7^6]$	Transitivity: [4.5.6.3]
Face symbol (Tile 1): $[4^3.5^6.7^6]$	Centroid (Tile 1): (0.333, 0.667, 0.609), Volume: 283.73 Å^3
Face symbol (Tile 2): $[6.7^2]$ (t-ukc)	Centroid (Tile 2): (0.815, 0.185, 0.891), Volume: 22.94 Å^3
Face symbol (Tile 3): $[6^8]$ (t-ber)	Centroid (Tile 3): (0.00, 0.00, 0.00), Volume: 166.07 Å^3

Table S2 Graphical representations of the tile-types of the interrupted framework structures listed in Table S1.

**$Cs_4CaSi_8O_{19}$**	
Tile 1: $[3^2.9^2.14^2]$	Tile 2: $[3^2.8^2.9^2.14^2]$
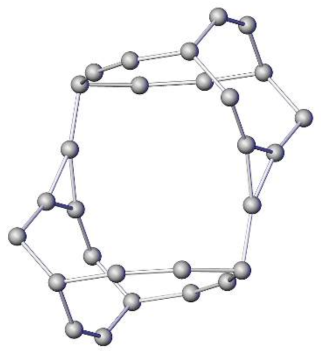	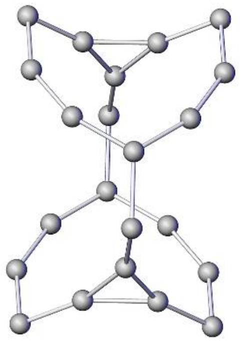
**Sarcolite**	
Tile 1: $[4^8.7^8.8^6]$	Tile 2: $[7^4]$
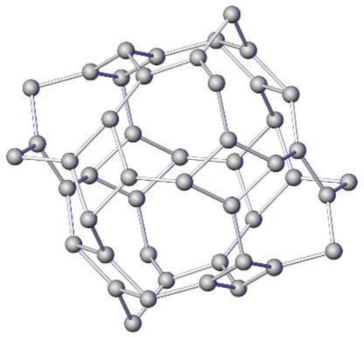	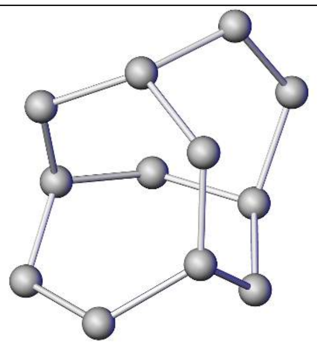
Tile 3: $[8^6]$	
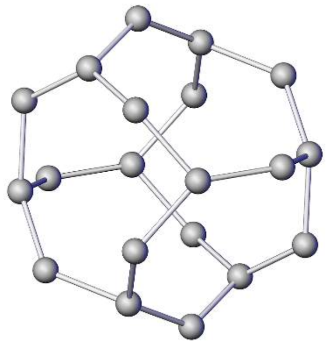	
**$Cs_2ErSi_6O_{14}F$**	
Tile 1: $[6^3]$	Tile 2: $[4^2.6^3.8^2.14^2]$

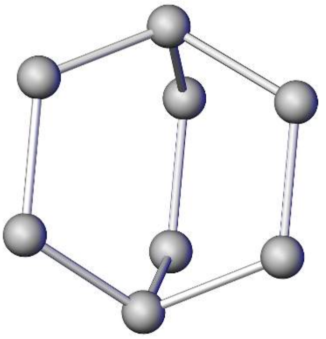	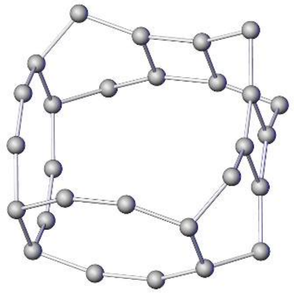
**Thornasite**	
Tile 1: [$4^2.6^2.12^2$]	Tile 2: [4^6]
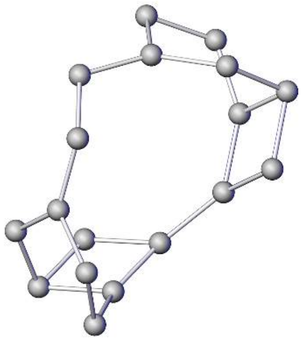	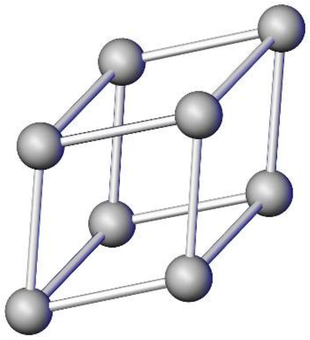
Tile 3: [6.14^2]	Tile 4: [$6.12.14^2$]
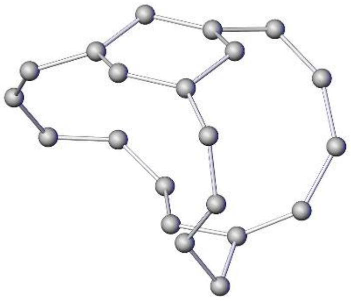	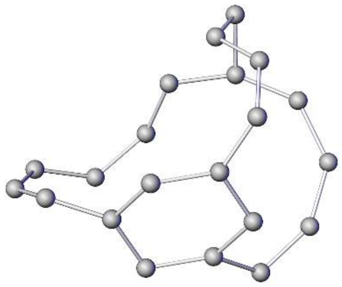
Tile 5: [10.14^2]	
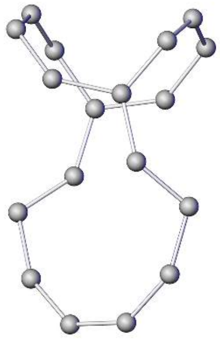	
**$K_3NdSi_7O_{17}$**	
Tile 1: [$5^4.14^2$]	Tile 2: [6^3]

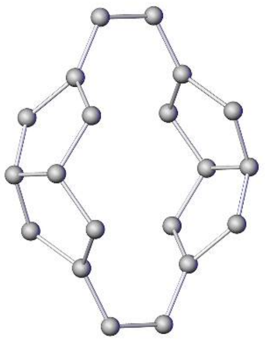	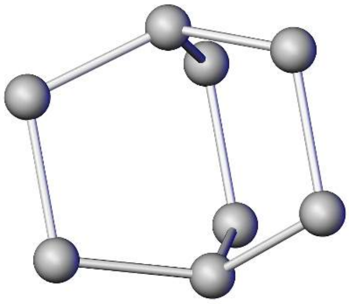
Tile 3: [$6^2.12^2.14^2$]	Tile 4: [$6^2.12^6$]
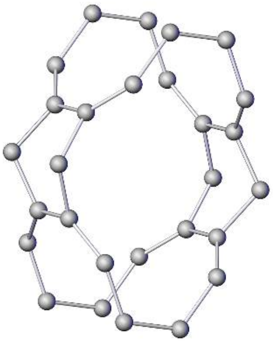	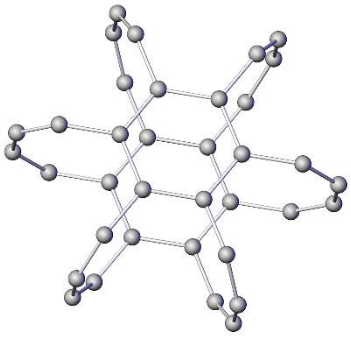
**Bavenite**	
Tile 1: [$4^2.8^2$]	Tile 2: [$6^{16}.8^2$]
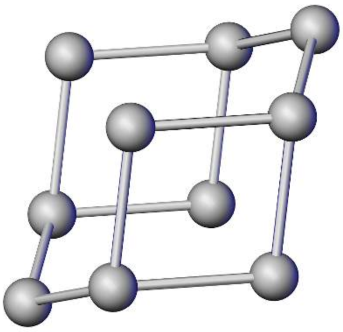	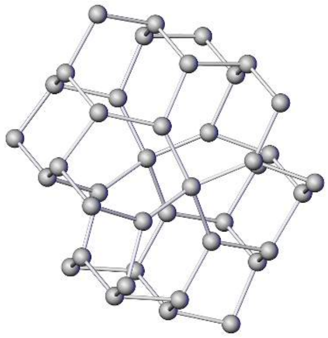
Tile 3: [6^4]	
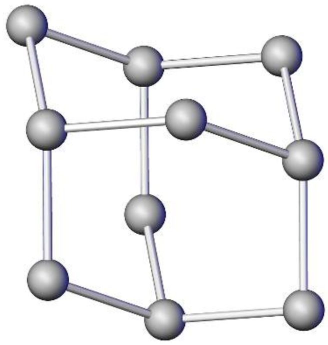	
**Maricopaite**	
Tile 1: [5^4]	Tile 2: [$5^6.8.12^2$]

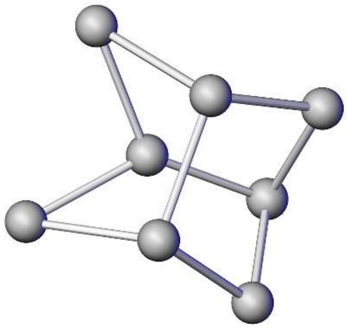	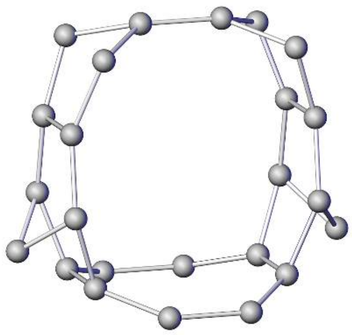
Tile 3: [$5^{10}.8.12^{2}$]	
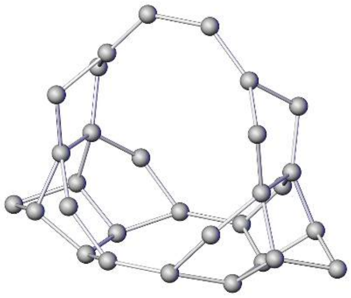	
**Leifite**	
Tile 1: [$4^{3}.5^{6}.7^{6}$]	Tile 2: [6.7^{2}]
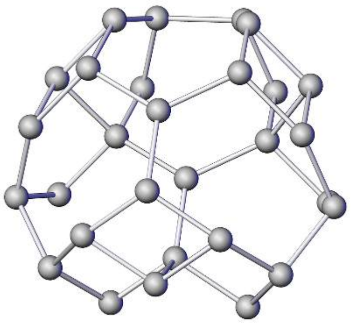	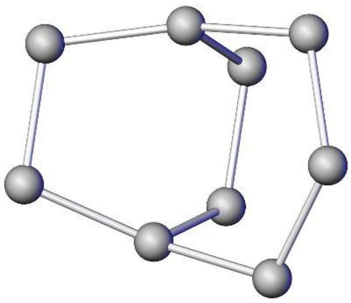
Tile 3: [6^{8}]	
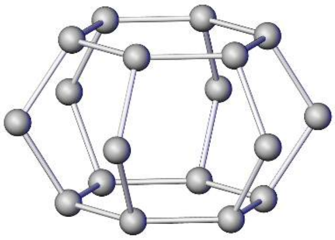	
